# From traditional fruit to modern functional food: chemical constituents, bioactive compounds, and therapeutic applications of Sukkari date palm (*Phoenix dactylifera*): a review

**DOI:** 10.3389/fnut.2025.1651121

**Published:** 2025-09-22

**Authors:** Rehab F. M. Ali, Ayman M. El-Anany

**Affiliations:** ^1^Department of Food Science and Human Nutrition, College of Agriculture and Food, Qassim University, Buraydah, Saudi Arabia; ^2^Special Food and Nutrition Department, Food Technology Research Institute, Agricultural Research Center, Giza, Egypt

**Keywords:** diet, Sukkari date palm, bioactive compounds, therapeutic applications, antioxidants

## Abstract

The Sukkari date palm (*Phoenix dactylifera L*.) is increasingly recognized for its potential in sustainable agriculture due to its nutritional benefits and low environmental impact. This cultivar thrives in arid conditions, requiring minimal water, which aligns with the growing demand for sustainable food sources. Sukkari dates are rich in essential nutrients, including dietary fiber, vitamins, and minerals, which support overall health. They possess bioactive compounds with antioxidant, anti-inflammatory, and antimicrobial properties, making them valuable in functional food development. The cultivation of Sukkari dates can enhance economic stability in arid regions, providing livelihoods and contributing to local economies. Innovative uses in biodegradable packaging and bioenergy align with circular economy principles, promoting eco-friendly practices. While Sukkari dates present numerous advantages for sustainable agriculture, challenges remain in optimizing postharvest technologies and scaling up byproduct utilization to fully realize their potential benefits. Future research should address these areas to enhance the sustainability of date palm cultivation.

## Introduction

The date palm, scientifically known as *Phoenix dactylifera*, is one of the oldest cultivated plants, offering a multitude of benefits across nutritional, environmental, economic, and ornamental domains. Its cultivation dates back to 5,500–3,000 BCE, and it remains a staple food source in arid regions (1). The date industry significantly contributes to the economies of producing countries, with global production reaching 8.53 million tons (1, 2). Dates are processed into various products, enhancing their market value and providing livelihoods for many communities (3). Date palms are also valued for their aesthetic appeal in landscaping and urban settings, contributing to biodiversity and enhancing the visual environment (4). The date fruit (*Phoenix dactylifera*) encompasses over 1,500 varieties, each with unique nutritional and pharmacological properties. Popular varieties such as Ajwa, Sukkari, and Ruthana are particularly noted for their health benefits, which stem from their rich composition of carbohydrates, vitamins, minerals, and antioxidants (5).

**Figure 1:**
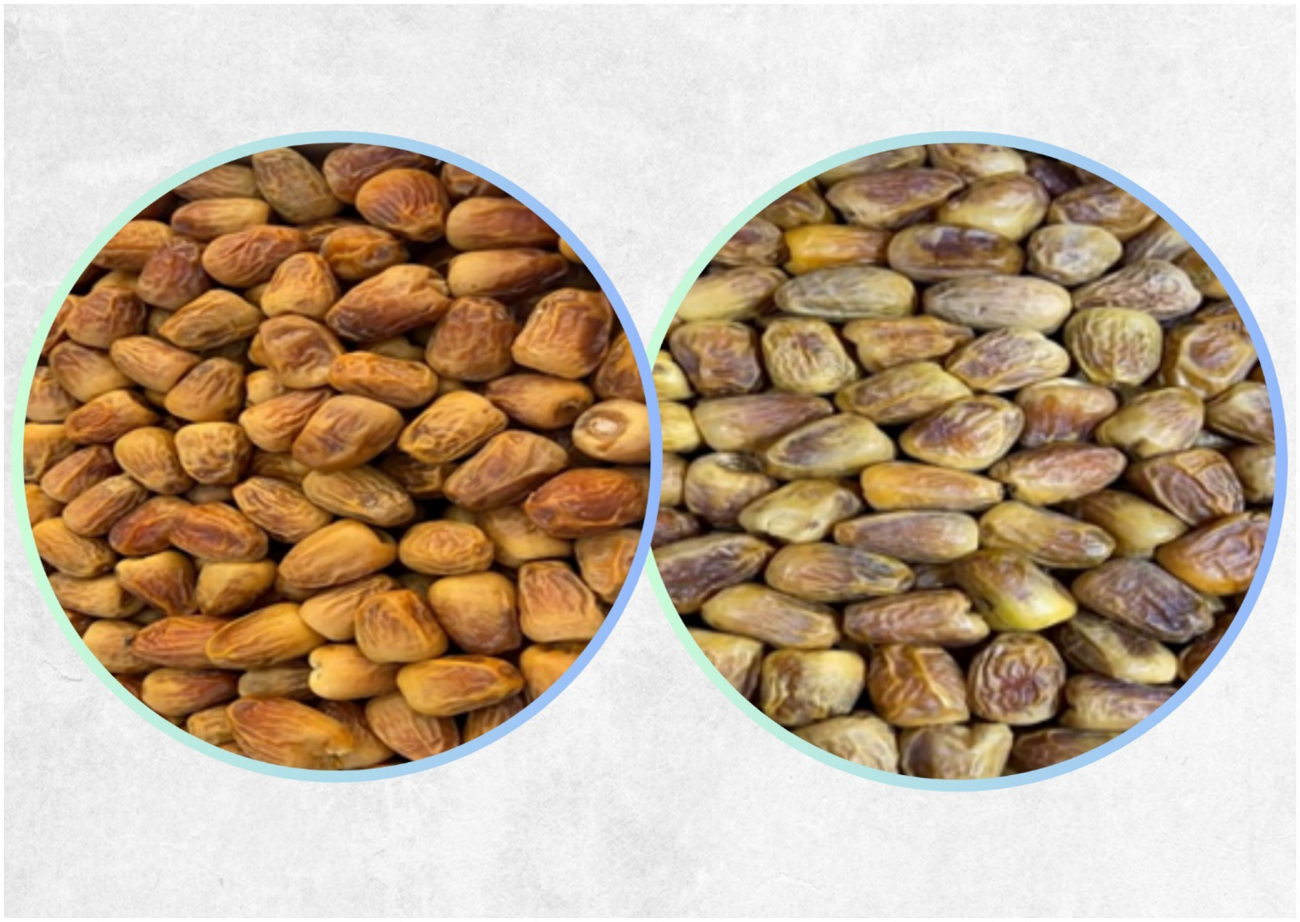
Sukkari dates soft and hard varieties.

Sukkari dates are a premium variety of dates, characterized by their soft texture and exceptional sweetness (Figure 1). The name “Sukkari” itself translates to “the sweet one” in Arabic, highlighting their well-known sweet flavor profile (6, 7). Grown predominantly in the fertile regions of Saudi Arabia, particularly in Al-Qassim, Sukkari dates have gained the nickname “royal dates” due to their premium quality and appeal. Sukkari dates feature a golden yellow to dark golden brown color with a soft, shiny skin. The dates are often plump and slightly oval-shaped, making them visually appealing. The flesh of Sukkari dates is moist and tender, often described as juicy and chewy. This texture is complemented by a unique caramel-like taste that many find irresistible. Sukkari dates are available in both soft and hard varieties, offering consumers options based on their texture preference. The soft variant is typically more sought after for its sumptuous quality (8). Unlike some date varieties that rely on freezing or processing to enhance their sweetness, Sukkari dates are naturally sweet, being enjoyed fresh without any added preservatives or treatments. Many people find Sukkari dates to be sweeter than other varieties, like Medjool dates, which adds to their allure, especially when paired with coffee or tea (8).

## Proximate composition of Sukkari dates

The proximate composition of Sukkari dates reveals a rich profile of sugars, minerals, and bioactive compounds, making them nutritionally significant. The predominant components include high sugar content, essential minerals, and fibers, which contribute to their health benefits (Table 1). Sukkari dates are characterized by a high sugar content, predominantly consisting of reducing sugars such as glucose (51.80%) and fructose (47.50%), while sucrose is found in only small quantities, indicating its role as a minor component in the overall sugar profile (9). Sukkari dates contain approximately 78.32% total sugars by dry weight (7). This composition highlights the nutritional value of Sukkari dates as a natural sweetener alternative. The high levels of reducing sugars contribute to the energy-boosting properties of Sukkari dates, making them suitable for athletes and as a natural sweetener in various food products (10). The Sukkari date, a popular variety from Saudi Arabia, exhibits a rich chemical composition that contributes to its nutritional and functional properties. This variety is characterized by high sugar content, essential minerals, and bioactive compounds, making it a valuable food source. The following sections detail the key components of Sukkari dates.

**Table 1:** Proximate composition of Sukkari dates.

**Constituents**	**Aljutaily et al. (11, 18) (g/100 g DW)**	**Alhomaid et al. (12) (g/100 g DW)**	**Siddeeg et al. (17) (g/100 g DW)**	**Hadi et al. (10) (% g DW)**	**Abdelbaky et al. (13) (% DW)**
Moisture	11.1	15.83	12.57	10.12	18.13
Protein	2.55	2.35	3.00	3.20	2.260
Fat	3.15	0.45	0.65	0.26	1.050
Ash	2.61	2.80	2.30	1.9	2.480
Carbohydrates	76.25	84.2	78.32	69.2	79.42
Fiber	4.35	10.2	3.15	3.77	2.130

DW means dry weight basis.

### Moisture

The moisture content of Sukkari dates varies significantly based on geographic origin, with reported values ranging from 11.1% to 18.13% on a dry weight basis. This variation is influenced by factors such as climate, soil conditions, and agricultural practices in different regions. In the Qassim Region of Saudi Arabia, moisture content ranges from 11.1% to 15.83% (11, 12). In Al-Madinah al-Munawwarah City, Saudi Arabia, values are higher, ranging from 14.5% to 18.13% (13, 256). For Sukkari dates from Iraq, results indicate moisture levels of 12.57% and 10.12% (7, 10). While the moisture content of Sukkari dates is crucial for quality and preservation, it is essential to consider that variations may also arise from post-harvest handling and storage conditions, which can further influence the final moisture levels in the product (14).

### Protein content

Sukkari dates, while often perceived as low in protein, exhibit a protein content ranging from 2.26% to 3.20% on a dry weight basis, indicating variability among different studies. This protein content, although modest compared to other food sources, can be enhanced when incorporated into various food products. The protein content of Sukkari dates is reported to be between 2.26% and 2.55% (11, 12). Comparatively, other date varieties show protein levels from 2.9% to 3.20% (7, 10, 256). Dates contain a variety of amino acids, including essential ones like glutamine and aspartic acid, which contribute to their nutritional profile (15). Sukkari dates can be utilized in high-energy and protein bars, where they serve as a nutrient-dense ingredient, enhancing the overall protein content of the product (16). Despite their relatively low protein content, Sukkari dates are rich in carbohydrates, dietary fiber, and essential minerals, making them a valuable component of a balanced diet. However, some may argue that their protein levels are insufficient for those requiring higher protein intake, suggesting a need for complementary protein sources in the diet (17).

### Fat content

The fat content of Sukkari dates exhibits variability, with reported levels ranging from 0.26% to 0.65% in several studies (7, 10, 12). However, some research indicates higher fat concentrations, such as 1.05% (13) and even 3.15% (11, 18). This discrepancy can be attributed to factors such as cultivar differences, ripening stages, and environmental conditions. Different date varieties exhibit distinct chemical compositions, affecting fat levels (19). The fat content can change as dates progress through their ripening stages, impacting nutritional profiles (17). Growing conditions, including soil and climate, can also influence the fat content in dates (19). While the majority of research indicates lower fat levels in Sukkari dates, the existence of higher fat percentages in certain studies highlights the need for further investigation into the factors contributing to these variations. This could lead to a better understanding of the nutritional value of dates and their potential health benefits.

### Ash content

The variations in ash content of Sukkari dates, ranging from 1.9% to 2.80%, can be attributed to several factors, including environmental conditions, cultivation practices, and the inherent characteristics of different date varieties. These factors influence the mineral composition and overall quality of the dates (7, 10–13, 18). The mineral content of the soil where the date palms are grown can significantly affect the ash content. Richer soils may lead to higher mineral accumulation in the fruit (20). The type and amount of fertilizers used can alter the mineral composition of the dates, leading to differences in ash content (21). Different irrigation techniques can influence the nutrient availability in the soil, which in turn affects the ash content of the dates. Variations in temperature and humidity can impact the growth and nutrient uptake of the date palms, thus affecting the ash content (20).

### Carbohydrate content

Sukkari dates are recognized for their high carbohydrate content, which ranges from 69.2% to 84.2%, making them a significant energy source. This carbohydrate composition primarily consists of natural sugars such as glucose and fructose, contributing to their sweet flavor and nutritional value (7, 10–13, 18). Sukkari dates contain approximately 78.32% carbohydrates on a dry weight basis, with glucose (51.80%) and fructose (47.50%) being the predominant sugars (7). The carbohydrate content varies among different date cultivars, with some reports indicating values as low as 69.20% in Sukkari dates (10). While Sukkari dates are an excellent source of carbohydrates and other nutrients, it is essential to consider their high sugar content, which may not be suitable for individuals managing blood sugar levels. Nonetheless, their low glycemic index makes them a viable option for many diets (22).

### Fiber content

The variations in fiber content of Sukkari dates, ranging from 2.1% to 10.2%, can be attributed to several factors including cultivar differences, ripening stages, and environmental conditions. These factors significantly influence the chemical composition of dates, including their dietary fiber content. Different cultivars of dates exhibit varying fiber levels; for instance, Sukkari dates show a range from 6.4% to 11.5 % fiber content depending on the specific variety analyzed (17). The study by Alhomaid et al. (12) identified the highest fiber content in a specific cultivar, highlighting the importance of genetic factors in fiber composition. In this regard, dates undergo four ripening stages (kimri, khalal, rutab, and tamer), which affect their nutritional profile, including fiber content. The fiber content can vary significantly at different ripening stages, contributing to the observed discrepancies in studies (19). Growing conditions such as soil quality, climate, and agricultural practices can impact the nutritional composition of dates (19). Postharvest handling and storage conditions also play a role in maintaining or altering the fiber content of dates (23). While the variations in fiber content are significant, it is also important to consider that the health benefits of dates, including their antioxidant properties and potential role in disease prevention, may not solely depend on fiber content but also on other bioactive compounds present in the fruit (24). In this regard, pectin is a water-soluble dietary fiber component found in date fruits, including the Sukkari variety (*Phoenix dactylifera* L.). Date fruits contain both soluble and insoluble dietary fiber, with soluble fiber representing a smaller but nutritionally significant portion. Research indicates that soluble dietary fiber (SDF) constitutes between 8.4% and 22.2% of total dietary fiber in various date fruit varieties (25). The main water-soluble dietary fiber components in dates include pectin, inulin, fructooligosaccharides, and β-glucan (26). Studies examining multiple date varieties found that total dietary fiber (TDF) values varied considerably, from 5.67%/100 g to 10.33%/100 g, with insoluble dietary fiber (IDF) constituting the bulk of dietary fiber content in all date fruit varieties (77.8% to 91.6%) (25). This indicates that pectin, as part of the soluble fraction, represents a relatively small but important component of the total fiber content.

The pectin content in dates varies significantly with ripening stage. Research shows that pectin content increases during fruit development, reaching maximum levels at the khalal stage (7.0–14.3%), then decreases at the tamer stage (1.3–1.9%) of maturity (27). This variation is attributed to enzymatic changes during ripening, where pectin methylesterase, polygalacturonases, and cellulase activity increase, leading to the breakdown of insoluble fibers into smaller soluble molecules (28). Pectin, as a soluble fiber, provides several health benefits. It is a natural complex heteropolysaccharide composed of galacturonic acid residues and various neutral sugars such as rhamnose, galactose, and arabinose (29). Pectin has been demonstrated to stimulate the release of GLP-1 (glucagon-like peptide-1), which can be employed in the treatment or prevention of metabolic syndrome, diabetes, and obesity (30). It promotes satiety and aids in controlling energy intake (31), reduces blood cholesterol by numerous strategies (32), and supports digestive health and good gut microbiota (33). The presence of pectin in Sukkari dates contributes to their functional food properties. Pectin's ability to moderate glycemic index, slow gastric transit, and promote satiety makes Sukkari dates potentially beneficial for metabolic health management (34). The fermentation of pectin by colonic bacteria generates short-chain fatty acids that contribute to favorable health outcomes (34).

## Mineral content of Sukkari dates

The mineral content of Sukkari dates is crucial for their nutritional value and health benefits (Table 2). These dates are rich in essential minerals, which contribute to their overall health-promoting properties. Sukkari dates contain high levels of potassium, calcium, and magnesium, which are vital for various bodily functions (7). They also provide trace minerals such as iron, zinc, and copper, which play roles in immune function and metabolic processes (35, 36). The potassium content in Sukkari dates can help regulate blood pressure, making them beneficial for cardiovascular health (35). Calcium and magnesium are essential for maintaining bone density and strength (7, 35). Sukkari dates are not only rich in minerals but also contain significant amounts of sugars, fiber, and antioxidants, enhancing their role as a functional food (10, 36). While Sukkari dates are nutritionally beneficial, it is important to consider that excessive consumption may lead to high sugar intake, which could counteract some health benefits, particularly for individuals managing blood sugar levels (24). Dates are a nutrient-dense fruit, exhibiting a high concentration of essential minerals such as iron, potassium, calcium, and magnesium, along with moderate levels of chloride, phosphorus, copper, sulfur, and silicon. The mineral content varies significantly among different date varieties, making them a valuable dietary source (35).

**Table 2:** Mineral content of Sukkari dates.

**Minerals**	**Aljutaily et al. (11, 18) (mg 100 g−1 FW)**	**Alhomaid et al. (12) (mg/Kg FW)**	**Siddeeg et al. (7) (mg/100 g DW)**	**Trabzuni et al. (37) (μg/g DW)**	**Hadi et al. (10) (% DW)**	**Abdelbaky et al. (13) (mg/100 g DW)**.	**Hamad et al. (38) (mg/100 g DW)**	**Ismail and Altuwairki (48) (%DW)**
Na	37.84	53.764	4.75	141.00	11.12	30.54	6.30	0.55%
Ca	108.23	629.16	186.55	819.00	65.41	64.12	0.512	0.35%
K	651.25	10,933	620.00	11,769.00	396.0	1,038.11	436.75	0.41%
P	47.95	724.608	26.50	598.00	77.1	109.92	80.640	0.10%
Mg	86.35	960.764	148.10	649.00	9.1	91.597	54.297	0.18%
Zn	9.37	1.33		14.17		1.16	1.077	1.9 ppm
Fe	4.46	2.33	6.50	2.10		6.11	1.64	91.8 ppm
Cu	2.28	1.189	1.20	20.31		0.37	3.94	1.8 ppm

### Sodium content

Sukkari dates exhibit a varied sodium content, with reported levels ranging from 4.75 to 14.1 mg per 100 g in several studies (7, 12, 37, 38), while higher concentrations of 30.54 and 37.84 mg per 100 g have been noted in more recent research (11, 13, 18). Despite these variations, the average sodium content in dates is relatively low, around 9 mg per 100 g, indicating that dates are a low-sodium food option (35). Sodium is crucial for several physiological functions, including muscle contraction and nerve signal transmission (39). While Sukkari dates provide some sodium, their overall contribution to dietary sodium intake is minimal compared to processed foods, which are the primary sources of sodium in modern diets (39). This highlights the importance of considering dietary sources when evaluating sodium intake and its health implications. Sodium is an essential nutrient, crucial for various physiological functions, including fluid balance, nerve transmission, and muscle function. While a daily intake of approximately 500 mg is necessary, excessive sodium consumption is linked to serious health issues such as hypertension, cardiovascular disease, and stroke. Dates, with a sodium content of only 3 to 10 mg per 100 g, provide a low-sodium option that can help mitigate these risks (36, 253). Foods like dates can help individuals maintain a healthy sodium intake, reducing the risk of hypertension and related diseases (40). Incorporating low-sodium options into diets can support public health initiatives aimed at reducing overall sodium consumption (39). In this regard, high sodium intake is associated with elevated blood pressure, increasing the risk of cardiovascular diseases and strokes (41, 42). A reduction of just 3 g of dietary salt could prevent tens of thousands of cardiovascular events annually (43).

### Calcium content

Calcium is a crucial mineral for various physiological functions, particularly in maintaining bone and dental health, facilitating muscle contractions, and supporting nerve signal transmission. Approximately 99% of the body's calcium is stored in bones and teeth, where it contributes to their strength and structure. Adequate calcium intake is essential to prevent deficiencies that can lead to conditions such as osteoporosis and osteomalacia, which are characterized by weakened bones and increased fracture risk (44). The body regulates calcium levels through hormones like parathyroid hormone and vitamin D, ensuring proper absorption and utilization (45). Deficiency can lead to osteoporosis, particularly in older adults, increasing fracture risk (46). Recommended daily intake varies, with higher needs during pregnancy and lactation (46). Calcium facilitates muscle contractions and is essential for heart function. It plays a critical role in neurotransmitter release and nerve signal transmission (44). The calcium content in Sukkari dates exhibits significant variability, influenced by factors such as cultivar and geographical region. Reports indicate a range from 62.91 mg to 186.55 mg per 100 g, with specific studies highlighting these differences. Alhomaid et al. (12) reported a calcium content of 62.91 mg per 100 g. Abdelbaky et al. (13) found a slightly higher value of 64.12 mg per 100 g. Moderate calcium levels were noted by Trabzuni et al. (37) at 81.9 mg and Aljutaily et al. (11, 18) at 108.23 mg per 100 g. The highest recorded calcium content was 186.55 mg per 100 g by Siddeeg et al. (7). Contrarily, Ismail and Altuwairki (48) reported an unusually high value of 350 mg per 100 g, which raises questions about measurement consistency. Dates are recognized for their rich mineral composition, including calcium, which is essential for bone health and metabolic functions (35). The variability in calcium content among different cultivars and regions suggests that Sukkari dates can be tailored for specific dietary needs, enhancing their role as a functional food (10).

### Potassium content

Potassium is crucial for maintaining intracellular fluid volume and the electrochemical gradient across cell membranes. The intracellular concentration of potassium is approximately 30 times greater than that in the extracellular space, a disparity maintained by the Na+/K+ ATPase pump. This gradient is vital for various physiological functions, including nerve conduction, muscle contraction, and kidney function (48). Potassium's role in repolarization is critical for muscle contraction, particularly in cardiac tissues, where imbalances can lead to arrhythmias (49, 50). While potassium is essential for numerous cellular processes, excessive or insufficient levels can lead to serious health issues, highlighting the importance of maintaining its balance in the body (49). The potassium content in Sukkari dates exhibits significant variability, with reported values ranging from 410 to 1,176.9 mg per 100 g. This variation is influenced by factors such as cultivar, ripening stage, and environmental conditions. Notably, Alhomaid et al. (12) and Trabzuni et al. (37) reported potassium levels of 1,093.3 and 1,176.9 mg per 100 g, respectively, while Ismail and Altuwairki (48) noted the lowest at 410 mg per 100 g. High potassium levels in dates can aid in managing hypertension and contribute to overall cardiovascular health (35). Dates are rich in various minerals, with potassium being a predominant element, essential for numerous physiological functions in both plants and humans (15, 51). While the potassium content in Sukkari dates is generally high, it is essential to consider the potential for variability based on cultivation and processing methods. This variability may influence dietary recommendations and health benefits associated with date consumption (15). Adequate potassium intake is linked to reduced risks of stroke and improved blood pressure regulation, especially in individuals with high sodium intake (52). Consuming 100 grams of dates can provide over 15% of the recommended daily allowance for potassium (53). While the benefits of potassium are well-documented, it is important to consider that excessive potassium intake can lead to hyperkalemia, particularly in individuals with kidney dysfunction. Thus, moderation is key in dietary consumption (54).

### Phosphorus content

Phosphorus is a crucial mineral in the human body, primarily involved in bone and teeth formation, energy metabolism, and cellular functions. It plays a significant role in skeletal development and maintenance, as well as in the phosphorylation processes that facilitate the utilization of carbohydrates and lipids. Additionally, phosphorus is essential for protein synthesis and ATP production, which is vital for energy supply in cells (55). Phosphorus is integral to ATP synthesis, the primary energy currency of the cell, facilitating energy transfer and storage (56). It also plays a role in the regulation of metabolic pathways through phosphorylation, impacting carbohydrate and lipid metabolism (57). Phosphorus is involved in cellular signaling and the regulation of protein functions through phosphorylation and dephosphorylation processes (58). It acts as a buffer in extracellular fluids, maintaining pH balance, which is crucial for various physiological processes (56). Conversely, while phosphorus is essential, excessive intake, particularly in conjunction with low calcium levels, may lead to adverse effects on bone health, highlighting the need for balanced dietary intake (59). The phosphorus content in Sukkari dates varies significantly, with reported concentrations ranging from 26.50 to 109.92 mg per 100 g. The lowest concentration of 26.50 mg was documented by Siddeeg et al. (7), while higher levels were noted by several studies, with moderate concentrations (47.95–80.64 mg per 100 g) reported by Aljutaily et al. (11, 18), Trabzuni et al. (37), Alhomaid et al. (12), and Hamad et al. (38). The highest phosphorus levels, reaching 100 and 109.9 mg per 100 g, were identified by Ismail and Altuwairki (48) and Abdelbaky et al. (13), respectively. Dates are recognized for their rich mineral content, including phosphorus, which contributes to their nutritional value (35).

### Magnesium content

Magnesium is an essential mineral that acts as a cofactor in over 300 enzymatic reactions, playing a crucial role in various physiological processes. Its involvement spans energy production, protein synthesis, and the regulation of muscle and nerve functions. Additionally, magnesium is vital for maintaining blood glucose levels and blood pressure, as well as supporting bone health and DNA synthesis (35, 60). Magnesium is integral to ATP utilization and transfer, essential for energy production through oxidative phosphorylation and glycolysis (60, 61). It facilitates over 300 enzymatic reactions, including those involved in protein synthesis and metabolic pathways (78). Magnesium aids in the active transport of calcium and potassium ions, which are crucial for nerve impulse transmission and muscle contraction (61, 62). Deficiencies can lead to neuromuscular symptoms such as muscle weakness and tremors (60). Magnesium plays a significant role in glucose metabolism, influencing insulin sensitivity and potentially reducing the risk of type 2 diabetes (62). It is also linked to cardiovascular health, with low magnesium levels associated with increased risks of hypertension and other cardiovascular diseases (78). Conversely, while magnesium is vital for numerous bodily functions, excessive supplementation can lead to adverse effects, including gastrointestinal disturbances and potential cardiovascular issues. Thus, maintaining a balanced intake is crucial for optimal health (62). The magnesium content in Sukkari dates varies significantly, with reported levels ranging from 54.29 to 180 mg per 100 g. This variation highlights the importance of specific cultivars and growing conditions in determining mineral content. Hamad et al. (38) and Trabzuni et al. (37) reported lower magnesium concentrations in Sukkari dates, specifically between 54.29 and 64.9 mg per 100 g. Aljutaily et al. (11, 18), Abdelbaky et al. (13), and Alhomaid et al. (12) documented moderate magnesium levels of 86.35, 91.59, and 96.07 mg per 100 g, respectively. This range indicates more favorable growing conditions or cultivar selection that enhances magnesium content. The highest recorded magnesium level of 180 mg per 100 g was noted by Ismail and Altuwairki (48). This peak suggests the potential for specific cultivars or optimal agricultural practices to significantly boost mineral content.

### Zinc content

Zinc is a crucial micronutrient that plays a significant role in various physiological processes, including immune function, protein synthesis, wound healing, DNA synthesis, and cell division. It is essential for normal growth and development during pregnancy, childhood, and adolescence. Zinc deficiency is a widespread issue that can lead to numerous health problems, highlighting the importance of maintaining adequate zinc levels through diet or supplementation (63). It plays a critical role in wound healing by participating in processes such as coagulation, inflammation, angiogenesis, and tissue formation (64). Zinc supports normal growth and development, particularly during pregnancy, childhood, and adolescence, by facilitating cell division and gene expression (65). It is concentrated in various body tissues, including bones and the pancreas, and is crucial for reproductive health and fertility (66). While zinc is essential for numerous bodily functions, it is important to note that the body does not have a specialized storage system for zinc, necessitating a regular dietary intake to maintain optimal levels (65). Additionally, the bioavailability of zinc in the diet can significantly impact its absorption, which is why strategies such as dietary diversification and supplementation are crucial in addressing zinc deficiency (63). The zinc content in Sukkari dates varies significantly, with recorded values ranging from 0.19 to 9.37 mg per 100 g. The lowest zinc concentration of 0.19 mg per 100 g was reported by Ismail and Altuwairki in 2016, while moderate values ranging from 1.07 to 1.41 mg per 100 g were noted by Alhomaid et al., Trabzuni et al., Abdelbaky et al., and Hamad et al. The highest and unusually high zinc concentration of 9.37 mg per 100 g was reported by Aljutaily et al. in 2022. The methods used in growing dates, including soil quality and fertilization, can significantly impact mineral content, including zinc (35). Variations in climate and geographical location can lead to differences in mineral uptake by date palms, affecting zinc levels (19). Different studies may employ varying techniques for measuring zinc content, leading to discrepancies in reported values (35). While the reported zinc content in Sukkari dates shows significant variation, it is important to consider the broader nutritional profile of dates. They are rich in other essential minerals and nutrients, which contribute to their health benefits. The unusually high zinc concentration reported by Aljutaily et al. (11) may warrant further investigation to understand the underlying causes and ensure consistency in future studies.

### Iron content

Iron is a crucial micronutrient that plays a vital role in various physiological functions, particularly in the formation of hemoglobin, which is essential for oxygen transport in the blood. The body requires iron not only for hemoglobin synthesis but also for energy production and immune function. Iron is a key component of hemoglobin, with approximately 70% of the body's iron bound to this protein in red blood cells (67). Hemoglobin facilitates oxygen transport from the lungs to tissues, and upon delivering oxygen, it binds carbon dioxide for exhalation (67). Iron is involved in energy metabolism, particularly in the electron transport chain within mitochondria, which is critical for cellular energy production (68). It also supports immune function, helping the body to fend off infections and recover from illnesses (69). The recommended daily intake of iron varies by age and gender due to differences in physiological needs. For adolescents, boys aged 14–18 require 11 mg per day, while girls in the same age group need 15 mg per day. Adult males (19+) require 8 mg, whereas adult females (19+) have a higher requirement of 18 mg per day, largely due to menstrual losses and increased iron needs during pregnancy (70, 71). Iron deficiency can lead to anemia, characterized by fatigue and a weakened immune response, affecting approximately 1.62 billion people globally (72). While iron is essential for health, excessive iron can lead to toxicity and related health issues. Thus, maintaining a balanced iron intake is crucial for optimal physiological function (73).

The iron content in Sukkari dates varies significantly, with reported concentrations ranging from 0.21 to 9.18 mg per 100 g. This variation highlights the nutritional value of dates as a source of iron, which is essential for preventing anemia and supporting overall health. Trabzuni et al. (37) reported the lowest iron concentration in Sukkari dates at 0.21 mg per 100 g. Hamad et al. (38) found a slightly higher minimum concentration of 1.64 mg per 100 g. Aljutaily et al. (11, 18) and Alhomaid et al. (12) documented moderate iron levels ranging from 2.33 to 6.50 mg per 100 g. Siddeeg et al. (7) and Abdelbaky et al. (13) corroborated these findings, indicating consistent moderate iron content across different studies. The highest concentration of iron, 9.18 mg per 100 g, was noted by Ismail and Altuwairki (48), suggesting that certain varieties or growing conditions may enhance iron accumulation. While the iron content in Sukkari dates is generally beneficial, it is essential to consider that excessive consumption of dates could lead to an imbalance in mineral intake, particularly if they are consumed in large quantities alongside other iron-rich foods. This highlights the need for moderation in dietary practices (74, 75).

In this regard, the iron concentration of dates, particularly Sukkari dates, is significant, with varied quantities found in different studies. Dates are known for their high mineral content, particularly iron, which can be used as a dietary supplement to treat iron deficiency. The bioaccessibility and bioavailability of iron in dates are regulated by a number of variables, including the presence of other nutrients that promote absorption (77). The bioavailability of iron in dates can be enhanced by combining them with vitamin C, which forms iron chelates that improve solubility and absorption (78). Studies indicate that the bioaccessibility of iron in food can vary widely, with some formulations showing only 0.23–2.52% bioaccessibility (79).

Non-heme iron, found in plant foods like dates, has a lower absorption rate (5–12%) compared to heme iron (14–18%) from animal sources (254). A varied diet that includes both animal and plant sources can improve overall iron status, as the presence of meat and certain fruits can enhance non-heme iron absorption (80, 81). Cooking process, fermentation and germination, can reduce inhibitors like phytic acid, thereby increasing iron bioavailability from foods like dates (82, 254). While dates can contribute to hemoglobin levels, their impact may be limited without a comprehensive dietary approach that includes iron-rich foods and methods to enhance absorption (83, 252). Iron supplementation and fortification may be necessary to achieve significant improvements in iron status, especially in populations with high deficiency rates (81).

### Copper content

Copper is an essential trace element that plays a critical role in various physiological processes, including enzyme function, energy production, and immune system support. It is integral to enzymes such as cytochrome c oxidase and superoxide dismutase, which are vital for cellular respiration and antioxidant defense, respectively (83, 84). Additionally, copper is involved in the synthesis of hemoglobin, connective tissue formation, and neurotransmitter biosynthesis (85, 86). The body regulates copper levels through transporters like ATP7A and ATP7B, which manage its absorption and distribution (84, 87). Copper acts as a cofactor for enzymes involved in respiration (cytochrome c oxidase) and antioxidant defense (superoxide dismutase) (83, 86). It is crucial for collagen synthesis and the maturation of connective tissues (84, 87). Copper supports neurotransmitter synthesis, impacting cognitive functions and mood (85). Deficiency can lead to fatigue, anemia, and weakened immune response (85). Insufficient copper may cause neurological issues, including coordination difficulties and numbness (83). Conversely, while copper is essential, excessive levels can lead to toxicity, resulting in oxidative stress and cellular damage, particularly in neurological contexts (83, 86). This duality highlights the importance of maintaining balanced copper levels for optimal health. The copper content in Sukkari dates varies significantly, with reported concentrations ranging from 0.18 to 3.94 mg per 100 g. This variation highlights the importance of monitoring heavy metal levels in food products, particularly dates, which are widely consumed. The lowest copper levels were reported at 0.18 mg by Ismail and Altuwairki (48) and 0.37 mg by Abdelbaky et al. (13). Several studies reported moderate copper levels, including 1.18 mg (12), 1.20 mg (7), 2.03 mg (37), and 2.28 mg (11, 18). The highest recorded copper concentration was 3.94 mg by Hamad et al. (38). Most studies indicate that copper levels in Sukkari dates generally fall within safe limits for consumption, although some samples may exceed recommended thresholds, necessitating regular monitoring (88, 89). Elevated copper levels can pose health risks, particularly if consumed in large quantities over time, emphasizing the need for awareness regarding heavy metal contamination in food (90).

Sukkari dates (*Phoenix dactylifera*) are recognized for their rich mineral content, which includes essential elements such as potassium, magnesium, copper, and selenium. These minerals contribute significantly to daily nutritional requirements, making Sukkari dates a valuable dietary component. However, while they provide beneficial nutrients, the levels of certain minerals must be monitored to ensure they align with recommended daily allowances. Sukkari dates are high in potassium and magnesium, which are crucial for various bodily functions. For instance, 100 g of dates can provide over 15% of the recommended daily allowance for these minerals (53). The presence of trace minerals like copper and selenium also supports metabolic processes, with Sukkari dates contributing to daily intake levels that are generally within safe limits (91). Despite their nutritional benefits, Sukkari dates may contain heavy metals such as lead and aluminum, which can exceed safe consumption levels. For example, lead levels in Sukkari dates were found to be concerning, with potential health risks associated with excessive intake (88). The estimated daily intake of lead from Sukkari dates surpasses the provisional tolerable daily intake, indicating a need for caution in consumption (88).

In contrast, while Sukkari dates are nutrient-rich, the potential for heavy metal contamination raises concerns about their safety as a regular dietary choice. This highlights the importance of sourcing dates from reputable producers to mitigate health risks.

The mineral content in Sukkari dates, such as calcium, magnesium, and zinc, varies significantly due to several factors, including environmental conditions, agricultural practices, and measurement techniques. This variability is not unique to Sukkari dates but is observed across different date varieties. The inconsistency in mineral levels can be attributed to factors such as soil quality, irrigation practices, and the specific cultivar of the date palm. These factors influence the mineral uptake and accumulation in the fruit, leading to the observed variations (35). Soil quality and irrigation practices significantly affect mineral uptake in dates. Variations in soil mineral content and water availability can lead to differences in the mineral composition of the fruit (35, 92). Geographical origin and agronomic traits, such as maturity period and fruit consistency, also play a role in mineral content variation. For instance, early ripening cultivars tend to have higher iron and calcium levels (92). Different analytical methods, such as inductively coupled plasma-mass spectrometry (ICP-MS) and atomic absorption spectrophotometry, can yield varying results in mineral content analysis. These methods have different sensitivities and detection limits, contributing to inconsistencies in reported mineral levels (91, 93). The unusually high concentration of minerals in Sukkari dates may indeed warrant further investigation, particularly regarding the techniques employed to determine mineral content. Variations in extraction methods and analytical techniques can significantly influence the reported mineral concentrations (35, 92). The SEM-EDS technique was used to analyze metal elements in date syrup, revealing significant differences in mineral content across varieties, including Sukkari (94). In the time ICP-AES method employed to assess mineral contents in various date fruits, this technique provided detailed insights into the concentrations of potassium, phosphorus, and other minerals in Sukkari dates (95). The method of extraction (e.g., hydraulic pressure vs. thermal) can lead to variations in mineral availability, as shown in the study of different date syrup extraction methods (94). Different date varieties exhibit distinct mineral profiles, which can contribute to the observed high concentrations in Sukkari dates (35, 95).

## Sugar profile of Sukkari date and glycemic indices

The sugar profile of Sukkari dates reveals a predominance of monosaccharides, specifically glucose and fructose, with lower levels of sucrose (Table 3). Research indicates that the sugar content in Sukkari dates varies slightly across studies but consistently highlights the dominance of glucose and fructose. The sugar content in Sukkari dates is approximately 78.32% of the dry weight, with glucose and fructose levels reported at 51.80 g and 47.50 g per 100 g dry weight, respectively, while sucrose is around 3.20 g per 100 g dry weight (7). This composition is consistent across various studies, with slight variations in the exact sugar content, such as 52.3 g of glucose and 48.2 g of fructose reported by Assirey (96). While the sugar profile of Sukkari dates is beneficial for energy and nutrition, it is important to consider the potential health implications of high sugar intake, particularly for individuals managing blood sugar levels. The evaluation of sugar types and amounts in 17 Saudi Arabian date cultivars revealed significant variations in sugar composition, primarily consisting of glucose, fructose, and sucrose. Among these cultivars, 13 exhibited high levels of glucose and fructose, indicating a preference for these sugars in date composition, while sucrose was either undetected or present in minimal concentrations. The glucose content ranged from 10.4 to 39.5 g per 100 g, and fructose from 8.2 to 36.1 g per 100 g. Notably, Sukkari dates contained glucose, fructose, and sucrose at levels of 10.4, 8.2, and 45.8 g per 100 g, respectively (251). While the findings highlight the predominance of glucose and fructose in Saudi date cultivars, it is essential to consider that the nutritional benefits of dates may also be influenced by other factors such as fiber content and antioxidant properties, which are crucial for overall health (7). The analysis of sugars in Sukkari dates reveals significant levels of glucose, fructose, and sucrose, contributing to their nutritional profile. Trabzuni et al. (37) reported glucose at 36.63 g, fructose at 35.23 g, and sucrose at 41.46 g per 100 g on a dry weight basis. This aligns with findings from other studies that highlight the sugar composition in various date varieties (37). While Sukkari dates are rich in sugars, some studies suggest that other date varieties may contain higher fructose levels, indicating a potential for varying health benefits and culinary uses across different cultivars (97). The analysis of sugars in Sukkari dates reveals significant levels of glucose, fructose, and sucrose, contributing to their nutritional profile. 10 reported glucose at 8.0 g, fructose at 6.2 g, and sucrose at 55.04 g per 100 g on a dry weight basis. This aligns with findings from other studies that highlight the sugar composition in various date varieties. In a study of 29 date varieties, Sukkari dates were among the few that contained 43.51% sucrose, while others predominantly had higher fructose levels (9). The analysis of sugar content in various date cultivars reveals significant variability in their composition, highlighting their nutritional richness. Hamad et al. (38) found that total sugar content in dates is notably high, with cultivars like Khla Al Qassim exhibiting substantial levels of glucose and fructose. Additionally, certain cultivars, such as Nabtit Ali and Sokary, were noted for their elevated sucrose concentrations. This diversity in sugar profiles underscores the potential health benefits and dietary significance of date fruits. Sukkari dates contain 1.5 mg/100 g FW glucose, 59.5 mg/100 g FW fructose, and 138.5 mg/100 g FW sucrose (38). They added that Nabtit Ali and Sokary have higher sucrose levels than other cultivars (38). The Sukkari date variety is characterized by its significant sugar content, with specific levels of glucose, fructose, and sucrose reported. Ismail and Altuwairki (48) noted glucose at 40.90 mg/g DW (4.9%), fructose at 72.27 mg/g DW (7.22%), and sucrose at 672.5 mg/g DW (67.25%). This composition highlights the Sukkari date's potential as a sweetener and its nutritional value. Other date varieties, such as Deglet Noor, also contain sucrose but generally have higher fructose levels (9). The total sugar content in various date fruits ranges from 61.7% to 78.6%, indicating variability among types (9). Despite the high sugar content, studies suggest that dates may not adversely affect blood glucose levels in individuals with type 2 diabetes (98).

**Table 3:** Sugar profile of Sukkari date flesh fruits (g/100 g dry weight).

**Sugars**	**Siddeeg et al. (7) (g/100 g DW)**	**Assirey (96) (g/100 g DW)**	**El-Mergawi et al. (251) (g kg−1 FW)**	**Trabzuni et al. (37) (g/100 g DW)**	**Hadi et al. (10) (%DW)**	**Hamad et al. (38) (mg/100 g FW)**	**Ismail and Altuwairki (48) (mg/g DW)**	**Zhang et al. (9) (g/100 g FW)**
Glucose	51.80	52.3	10.4	6.76	8.0	1.5	40.90	10.07
Fructose	47.50	48.2	82	6.50	6.2	59.5	72.27	10.09
Sucrose	3.20	3.2	458	7.65	55.04	138.5	672.5	43.51

The glycemic index of dates varies, with some studies indicating no significant impact on glycemia (98). Conversely, while Sukkari dates are rich in sugars, their consumption should be moderated, especially for individuals managing blood sugar levels, as excessive intake could lead to glycemic spikes despite their potential health benefits. The evaluation of glycemic indices (GI), glycemic load (GL), and glycemic response of various date varieties reveals significant insights into their nutritional impact, particularly for individuals managing blood glucose levels. AlGeffari et al. (100) identified that the Shaqra, Sukkary, and Sag'ai varieties exhibited low GIs, making them suitable for consumption by those concerned about glycemic control. The classification of GIs into into low (< 55), medium (56–69), or high (≥70) categories aids in understanding their potential effects on blood sugar levels. Shaqra (42.8), Sukkary (43.4), and Sag'ai (44.6) are categorized as low GI, indicating a slower glucose release into the bloodstream (100). The ripeness of dates significantly affects their GI and GL. For instance, the Tamer stage generally shows lower GIs compared to the Khalal stage (101). The GL varies across maturation stages, with the Rutab stage presenting the highest GL values (101). While the findings suggest that certain date varieties can be beneficial for glycemic control, some studies indicate that the overall impact of dates on blood glucose may not be as detrimental as previously thought. For instance, Sachdev and Misra (98) highlight that moderate consumption of dates does not significantly impair glycemic levels in individuals with type 2 diabetes. This suggests a need for further research to clarify the role of dates in diabetic diets. High consumption of date fruits can influence blood sugar levels, necessitating careful management of their intake. Dates contain a significant amount of fructose, which is sweeter than glucose and may promote satiety, potentially mitigating rapid blood sugar spikes. Research indicates that certain date varieties exhibit low glycemic indices (GI), suggesting they may be suitable for individuals with diabetes when consumed in moderation. Dates have a GI ranging from 35.5 to 74.6, depending on the variety and ripeness (95 and 97). Studies show that consumption of dates does not significantly impair glycemic control in type 2 diabetes patients, with some evidence indicating a reduction in fasting and postprandial glucose levels (102, 103). Moderate consumption of dates (2–3 servings per day) is generally recommended for diabetic patients, as they can be part of a balanced diet without significant adverse effects on blood glucose levels (99 and 100). Healthcare providers should consider the ripeness and variety of dates when advising patients, as these factors influence their glycemic response (100). Conversely, while dates can be beneficial, excessive consumption may still pose risks for blood sugar spikes, particularly in individuals with poor glycemic control. Therefore, personalized dietary advice is crucial for managing diabetes effectively. Studies show that moderate consumption of dates (60 g daily) does not significantly worsen glycemic control in type 2 diabetes patients (104). Despite their benefits, excessive date consumption can lead to increased blood sugar levels, especially in individuals with poor glycemic control (105). Individual responses to date consumption can vary, necessitating personalized dietary recommendations to mitigate risks. While dates can be a healthy addition to a diabetic diet, their high sugar content requires careful management. Individuals should consult healthcare professionals to tailor their dietary choices effectively (98). The glycemic index (GI) of dried dates has been shown to be relatively high, indicating a significant impact on blood glucose levels. In a study involving a 50-gram oral glucose tolerance test (OGTT) and an oral dried dates tolerance test, the GI of dried dates was calculated at 72%, which increased to 89% when the portion was halved (106). This suggests that even small amounts of dried dates can lead to considerable glycemic responses, highlighting their potential implications for dietary management, particularly for individuals with diabetes (107). High GI foods can lead to rapid increases in blood glucose, which is critical for diabetic dietary planning (108). The consumption of dried dates may necessitate careful monitoring of blood glucose levels, especially in diabetic patients (109).

Dried dates are nutrient-dense but can pose challenges for blood sugar control due to their high GI. The balance between enjoying the nutritional benefits of dates and managing glycemic responses is essential for health (110).

The low glycemic index (GI) of dates can be attributed to their unique composition, particularly the presence of dietary fiber, such as pectin, and phenolic compounds. These components contribute to the delayed digestion and absorption of carbohydrates, resulting in a more gradual increase in blood glucose levels (111, 112). Dietary fiber, including pectin, enhances the viscosity of the intestinal contents, which slows down glucose metabolism and reduces enzyme–substrate interactions (113, 114). Studies indicate that the consumption of dates leads to lower postprandial glucose excursions, particularly in individuals with type 2 diabetes, suggesting that fiber plays a crucial role in glucose regulation (103). Dates are rich in phenolic compounds, which not only contribute to their low GI but also provide antioxidant benefits, potentially improving overall health (115, 116). Phenolic compounds in dates may inhibit digestive enzymes, further contributing to lower glucose absorption rates (113, 114). The combination of dietary fiber and phenolics can enhance glucose adsorption and modulate gut microbiota, promoting better glycemic control (113, 114). In contrast, while the low GI of dates is beneficial, it is essential to consider that excessive consumption of any carbohydrate source, even those with low GI, can still lead to elevated blood glucose levels if not balanced within a diet (105). Studies indicate that moderate consumption of dates does not significantly impair glycemic control in individuals with type 2 diabetes (116). Low-GI diets, including dates, can improve insulin sensitivity and lipid profiles, contributing to better overall metabolic health (117).

Despite their low GI, dates are high in sugars (over 70%), which can lead to elevated blood glucose levels if consumed in excess (116). A balanced diet is essential; even low-GI foods can cause spikes in blood sugar when not moderated (118). While low-GI foods like dates can support glycemic control, it is vital to maintain moderation and balance within the overall diet to prevent potential adverse effects on blood glucose levels.

## Nature antioxidants of Sukkari dates

The antioxidant properties of glutathione (GSH), ascorbic acid (ASC), and tocopherol are crucial for plant health, particularly in date palm cultivars. Variations in their concentrations among different cultivars highlight the genetic and environmental influences on antioxidant levels (38). GSH levels varied significantly across date palm cultivars, ranging from 0.011 to 0.295 μmol·g^−1^ FW. The highest GSH concentrations were found in Rashodia (0.247), Khlas Al Ahsa (0.177), and Nabtit Ali (0.295), while Khodry had the lowest at 0.011 μmol·g^−1^ FW. In this regard, Sukkari dates had GSH at levels of 0.059 μmol·g^−1^ FW (38). ASC content also showed considerable variation, from 0.051 to 0.541 μmol·g^−1^ FW. Rashodia (0.541), Sokary (0.526), and Nabtit Ali (0.516) exhibited the highest ASC levels, whereas Ajwa Al Madinah had the lowest at 0.051 μmol·g^−1^ FW. Total tocopherol content ranged from 0.09 to 0.28 μmol·g^−1^ FW, with Sokary having the highest concentration (0.28) and Khla Al Qassim the lowest (0.09) (38). The variations in antioxidant levels among cultivars suggest that genetic factors and environmental conditions significantly influence the antioxidant capacity of date palms, which is essential for their resilience against oxidative stress (119). Dates are rich in carbohydrates, dietary fiber, and essential vitamins, contributing to various health benefits such as antioxidant and anti-inflammatory properties (120). The presence of vitamins supports metabolic functions and overall health, making dates a valuable dietary component (121). The Sukkari date palm fruit is rich in vitamins, with vitamin E being the most abundant at 19.74 mg/g, highlighting its antioxidant properties (122), followed by vitamin B at 2.38 mg/g. Other B vitamins, including B1, B3, B7, B9, and B12, are present in smaller amounts. This aligns with previous findings that dates contain a variety of vitamins, including vitamin C, thiamine (B1), riboflavin (B2), niacin (B3), and vitamin A (47). The nutritional profile of dates, including their vitamin content, contributes to their health benefits and popularity as a food source (Table 4).

**Table 4:** Organic acids, nature antioxidant, and vitamins of Sukkari dates.

**Organic acids**	**Hamad et al. (38) (mg/100 g FW) (mg·g−1 FW)**
Oxalic acid	2.18
Malic acid	10.43
Succinic acid	9.26
Citric acid	4.65
Isobutyric acid	2.94
Formic acid	0.29
Nature antioxidant	Hamad et al. (38)
GSH (μmol·g−1 FW)	0.059
GSH Redox Status (%)	21.736
Ascorbate (μmol·g−1 FW)	0.526
Ascorbate Redox Status (%)	87.570
Alfa tocopherols (ng/100 g FW)	0.218
Beta tocopherols (ng/100 g FW)	0.019
Gamma tocopherols (ng/100 g FW)	0.043
Delta tocopherols (ng/100 g FW)	0.011
Vitamins (mg/g DW)	Ismail and Altuwairki (48)
B1	0.55
B2	ND
B3	0.40
B5	ND
B6	2.38
B9	0.05
B12	0.55
Vitamin E	19.74
Vitamin A	ND

Ascorbate is known for its role in enhancing immune function and reducing oxidative stress, which can lead to chronic diseases (123). The ascorbate content of Sukkari dates, measured at 0.526 μmol·g^−1^ FW, highlights the nutritional significance of this fruit. Ascorbate, or vitamin C, is a crucial antioxidant that plays various roles in plant physiology and human health (38). Dates, including Sukkari, are rich in essential nutrients, with ascorbate contributing to their antioxidant properties (19). The accumulation of ascorbate in fruits is influenced by factors such as cultivar, ripening stage, and environmental conditions (257). Sukkari dates, like other varieties, may exhibit varying ascorbate levels depending on these factors, impacting their health benefits. The presence of ascorbate in dates supports their classification as functional foods, promoting overall health and wellbeing (124).

Ascorbate is vital for plants under abiotic stress, helping to regulate redox homeostasis and mitigate oxidative damage. It participates in the ascorbate–glutathione cycle, enhancing the plant's ability to cope with environmental stressors (125). The ascorbate redox status of Sukkari dates, reported at 87.570%, highlights the significant role of ascorbate (vitamin C) in maintaining cellular redox homeostasis and antioxidant defense (38). This high percentage indicates a robust capacity for scavenging reactive oxygen species (ROS), which is crucial for plant health and stress response. Ascorbate acts as a cofactor for over 60 dioxygenases, influencing gene expression and cellular functions. It serves as a major antioxidant, effectively neutralizing ROS and reducing Fe(III) for iron uptake (126).

The bioactive compounds in dates, including tocopherols, contribute to their health benefits, such as anti-inflammatory and antioxidant effects (127). The main forms include alpha, beta, gamma, and delta tocopherols, each with distinct biological activities. Tocopherols are associated with various health benefits, including potential cancer prevention, although results can vary significantly among different tocopherol types (128). α-Tocopherol is known for its antioxidant capabilities, which help protect cells from oxidative stress and may reduce the risk of chronic diseases (127). The α-tocopherol content in Sukkari dates is reported to be 0.218 ng/100 g fresh weight (38), indicating a relatively low concentration of this essential vitamin E form. While Sukkari dates contain α-tocopherol, other sources like nuts and vegetable oils typically have higher concentrations, emphasizing the need for a varied diet to meet vitamin E requirements (129). Tocopherols, including beta tocopherol, are essential antioxidants that contribute to human health by protecting against oxidative stress and various diseases (130). The beta tocopherol content in Sukkari dates is reported to be 0.019 ng/100 g fresh weight (FW) according to Hamad et al. (38). Sukkari dates exhibit a low concentration of beta tocopherol (131). Other tocopherols, such as α-tocotrienol and γ-tocopherol, are present in higher amounts in different date varieties, indicating variability in tocopherol profiles across species (131, 132). The low levels of beta tocopherol in Sukkari dates suggest that while they are nutritious, they may not be a significant source of this particular vitamin E form compared to other foods (133). Gamma tocopherols are recognized for their ability to neutralize free radicals, potentially reducing oxidative stress and associated diseases (133). The gamma tocopherol content in Sukkari dates, reported at 0.043 ng/100 g fresh weight (FW) (38), highlights the nutritional profile of this date variety. Gamma tocopherols are part of the vitamin E family, known for their antioxidant properties and health benefits (133). The delta tocopherol content in Sukkari dates is reported to be 0.011 ng/100 g fresh weight (FW) according to Hamad et al. (38). This finding highlights the presence of tocopherols, which are important antioxidants, in various food sources, including dates. Tocopherols, particularly alpha and gamma, are known for their ability to inhibit oxidation processes in lipids, which is crucial for food preservation and health benefits (134).

## Phenolic content and antioxidant properties of Sukkari dates

The Sukkari date variety exhibits notable levels of total phenolic content (TPC), total flavonoid content (TFC), and antioxidant activities, making it a significant source of bioactive compounds. Research indicates that Sukkari dates possess a high concentration of phenolic compounds, which are crucial for their antioxidant properties (Table 5). The presence of phenolic compounds and flavonoids contributes to their antioxidant capacity (135). Anti-inflammatory Effects: Regular consumption may help reduce inflammation and lower the risk of chronic diseases (115). Fruits, particularly those from the date palm (*Phoenix dactylifera*), are recognized for their significant health benefits, primarily due to their rich antioxidant content. These antioxidants, including vitamins C and E, carotenoids, and polyphenols, play a crucial role in protecting the body from oxidative stress, which can lead to various diseases. Recent studies highlight the date palm's ability to scavenge free radicals, showcasing its potential as a functional food (136). Date fruits exhibit strong radical scavenging activity, with various extracts showing different levels of effectiveness. For instance, the ethyl acetate extract of Sukkari dates demonstrated the highest antioxidant potential (13). The presence of polyphenolic compounds correlates with their ability to inhibit oxidative damage, supporting their role in disease prevention (137, 138). Regular consumption of dates is associated with anti-inflammatory, anticancer, and immune-stimulant effects, making them a valuable addition to the diet (13, 135). Studies indicate that a diet rich in fruits, including dates, can significantly reduce the risk of chronic diseases such as cancer and cardiovascular issues (137).

**Table 5:** Total Phenolic content, total flavonoid content, and antioxidant activities of Sukkari dates.

**Parameter**	**Zihad et al. (139)**	Aljutaily et al. (11, 18)^*^	**Siddeeg et al. (7)**	**El-Mergawi et al. (251)**	**Trabzuni et al. (37)**	**Hamad et al. (38) (mg/100 g DW)**	**Ismail and Altuwairki (48) DW**	**Abdelbaky et al. (13)**
TPC	39.01 (mg GAE/g DW)	54.12 0 (mg GAE g^−1^)	62.50 mg GAE/100 g; 0.6 mg GAE g^−1^	2.48 (g kg^−1^ as gallic acid FW); 480 mg GAE g^−1^	25.90 (mg GAE/100 g extract DW); 0.25 mg GAE g^−1^	17.10 [El-Mergawi et al., (251) DW]; 0.1 mg GAE g^−1^	7.00 (mg eq GA/g extract)	29.61 (mg GAE·g^−1^ Extract)
TF	61.03 mg QE/g of dry extract	56.79 (mg QE g^−1^)	3.20 mg CE/100 g; 0.32 mg QE g^−1^	–	4.86 (mg QE/100 g extract); 0.48 mg QE g^−1^	1.983 (mg/100 g DW); 0.019 mg QE g^−1^	0.697 (mg eq Qu/g extract)	17.40 (mg RE·g^−1^ Extract)
DPPH	176.9 (μg/mL)	87.15 (μmol of TE g^−1^)	43–76%	4.12 μmol Trolox g^−1^ FW	88%	14%	–	–
ABTS-RSA [μmol of TE g−1]	–	96.18	47–60 %	–	–	–	–	–

While the health benefits of date fruits are well-documented, it is essential to consider that excessive consumption of high-sugar fruits may lead to adverse effects, particularly for individuals with diabetes or metabolic disorders. Balancing fruit intake with overall dietary needs remains crucial for optimal health. The total phenolic content (TPC) of Sukkari dates exhibits a wide range, reflecting variations in extraction methods and sample conditions. Studies indicate that TPC can range from as low as 0.17 mg/g DW to as high as 480 mg/g DW, with some reports noting even higher concentrations in different contexts. Hamad et al. (38), Trabzuni et al. (37), and Siddeeg et al. (7) reported TPC values between 0.17 and 0.6 mg/g, indicating significant variability in phenolic content based on cultivar and extraction methods. Zihad et al. (139) and Aljutaily et al. (11, 18) found moderate TPC levels ranging from 39.01 to 54.12 mg/g DW, suggesting that certain conditions or cultivars may enhance phenolic extraction. El-Mergawi et al. (251) reported exceptionally high TPC levels of 480 mg GAE/g, highlighting the potential for specific extraction techniques to yield higher phenolic concentrations. The variability in TPC among Sukkari dates underscores the importance of extraction methods and cultivar selection in maximizing health benefits. However, it is essential to consider that while higher phenolic content is often associated with greater antioxidant activity, the bioavailability and efficacy of these compounds can vary significantly, suggesting a need for further research into their practical applications in nutrition and health.

Flavonoids, a diverse group of polyphenolic compounds found abundantly in fruits, including date fruits, play a significant role in promoting health and preventing diseases. Their antioxidant properties help combat oxidative stress, which is linked to various chronic diseases (140). Flavonoids are known for their ability to scavenge free radicals, thereby reducing oxidative stress and preventing cellular damage (141, 142). Regular consumption of flavonoid-rich fruits is associated with a lower risk of cardiovascular diseases, cancer, and neurodegenerative disorders (141, 143). Flavonoids exhibit anti-inflammatory properties, which can mitigate chronic inflammation linked to various health issues (143, 144). Date fruits are particularly rich in flavonoids and polyphenols, contributing to their health-promoting effects. The organoleptic properties of dates, combined with their health benefits, make them a popular choice among consumers seeking nutritious options (141). The flavonoid content in Sukkari dates exhibits a wide range, with concentrations varying significantly across studies. This variability can be attributed to factors such as extraction methods, environmental conditions, and the specific cultivar of dates analyzed. Studies reported lower flavonoid levels, such as 0.019 mg QE g^−1^, 0.32 mg QE g^−1^, 0.48 mg QE g^−1^, and 0.697 mg eq Qu/g extract indicating a baseline for Sukkari dates (7, 37, 38, 47). A moderate concentration of 17.40 mg RE·g^−1^ was noted by Abdelbaky et al. (13), suggesting that extraction methods can influence flavonoid yield. Notably, higher concentrations were recorded, with Zihad et al. (139) reporting 56.79 mg QE g^−1^ and Aljutaily et al. (11, 18) documenting 61.03 mg QE g^−1^, highlighting the potential for significant bioactive compound extraction from Sukkari dates. The variability in flavonoid content underscores the importance of extraction techniques and environmental factors in determining the nutritional profile of Sukkari dates. However, it is also essential to consider that not all studies may have accounted for the same variables, which could lead to discrepancies in reported values.

The DPPH assay is a widely used method for evaluating antioxidant activity due to its simplicity and effectiveness (145). It measures the ability of compounds to donate protons to neutralize free radicals, with lower IC50 values indicating higher antioxidant capacity (146). Date fruits (*Phoenix dactylifera*) are recognized for their rich phenolic content, which significantly contributes to their antioxidant capacity. Various studies indicate that different date varieties exhibit substantial variations in phenolic levels, correlating with their antioxidant activities (124, 147). The DPPH assay results demonstrate that date extracts can effectively scavenge free radicals, with activities reported between 54.43% and 80.89% (148, 250). The DPPH assay reveals that date extracts can scavenge free radicals effectively, with varying efficiencies across different varieties. For example, the Sukkari variety showed an IC50 value of 132.4 μg/mL, indicating strong antioxidant potential (13). The antioxidant activities of date extracts are often higher in certain varieties, such as Khalas and Khenaizi, which demonstrated significant DPPH scavenging capabilities (148). The high levels of phenolic compounds in dates suggest their potential as natural antioxidants, which could replace synthetic alternatives in food and pharmaceutical industries (149). While the antioxidant properties of date fruits are well-documented, it is essential to consider that the health benefits may vary based on individual consumption patterns and the specific varieties consumed. Further research could explore the long-term health impacts of regular date consumption.

The study by Zihad et al. (139) highlights the antioxidant potential of date palm cultivars using the DPPH free radical scavenging assay, revealing that the Safawy cultivar exhibits the highest scavenging activity. This method is recognized for its sensitivity and reliability in assessing antioxidant properties, making it a preferred choice in various studies. The findings suggest that date palm extracts possess significant proton-donating capacity, classifying them as primary antioxidants. In Zihad et al.'s study, the IC50 values for Safawy, Ajwah, and Sukkari were 104, 125, and 177 μg/mL, respectively, all demonstrating good scavenging activity compared to ascorbic acid (IC50 12.09 μg/mL). The antioxidant properties of date palms can contribute to health benefits by mitigating oxidative stress, which is linked to various diseases (150). Natural antioxidants from plant sources, like date palms, are increasingly preferred over synthetic alternatives due to their safety and efficacy (151). The results from Aljutaily et al. (11, 18) indicate that Sukkari dates exhibit significant antioxidant activity, with DPPH-RSA measured at 87.15 ± 5.64 μmol of TE g−1 and ABTS-RSA at 96.18 ± 4.98 μmol of TE g−1. This highlights the potential of date fruits as natural antioxidants, which can be further understood through various aspects. Date fruits, particularly from the Sukkari variety, are rich in phenolic compounds, which contribute to their antioxidant properties (13).

The DPPH radical scavenging activity of Sukkari date palm flesh extracts has been evaluated, revealing significant antioxidant potential. The ethanolic and methanolic extracts demonstrated scavenging capacities of 43–76% and 40–72%, respectively, with IC50 values of 309.75 and 389.23 μL equivalent/mL, indicating a moderate ability to neutralize free radicals compared to ascorbic acid, which served as a reference compound. The ethanolic extracts exhibited higher antioxidant activity, although the differences were statistically non-significant (7). These findings suggest that Sukkari date palm fruit contains diverse bioactive components that may protect biological macromolecules from oxidative damage.

The antioxidant activity of various date cultivars demonstrates significant variability, influenced by genetic factors and phenolic content. In a study measuring 17 date cultivars, antioxidant activity ranged from 2.46 to 9.80 μmol Trolox g^−1^ FW using the DPPH method and from 5.90 to 17.78 μmol Trolox g^−1^ FW with the FRAP method. Notably, cultivars such as Nabtat-Ali exhibited the highest antioxidant values, while Osilah showed the lowest. Sukkari dates had 4.12 μmol Trolox g^−1^ FW (251). A strong correlation exists between antioxidant activity and total phenolic content across cultivars (152). Higher phenolic concentrations generally lead to increased antioxidant capacity. Genetic factors significantly influence the antioxidant profiles of date cultivars, leading to diverse antioxidant activities (153). While the antioxidant potential of date cultivars is promising, it is essential to consider that environmental factors and ripening stages can also affect these values, potentially leading to variations in antioxidant activity not solely attributable to genetic differences (154). The DPPH free radical scavenging activity of various date palm fruit extracts demonstrates significant antioxidant potential, with notable differences among varieties. Research indicates that Khlas El Shiokh, Khlas Al Ahsa, and Khlas Al Kharj exhibit the highest scavenging capacities, exceeding 38%, while the Sokary cultivar shows a lower capacity of 14% (38). This variability is attributed to the phenolic and flavonoid content present in the extracts, which correlates with their antioxidant activities. While the antioxidant properties of date varieties are promising, it is essential to consider that factors such as extraction methods and environmental conditions can influence these results, potentially leading to variations in antioxidant capacity across studies.

The fractionation of phenolic and flavonoid compounds in Sukkari dates involves the separation and analysis of these bioactive compounds, which are known for their antioxidant properties (Table 6). The rich phenolic and flavonoid content in dates positions them as valuable sources for functional food additives and nutraceuticals. The antioxidant properties of these compounds can potentially replace synthetic antioxidants in the food and pharmaceutical industries (149). The caffeic acid content in Sukkari dates, as reported in various studies, ranges from 0.019 to 5.40 mg/100 g dry weight (DW). This variation in caffeic acid content can be attributed to several factors, including the extraction method, the specific date variety, and the environmental conditions under which the dates are grown. The phenolic content, including caffeic acid, plays a significant role in the antioxidant properties of dates, which are highly valued for their health benefits (38, 155, 156). The variation in caffeic acid content can be influenced by the ripening stage of the dates, as phenolic content changes during the ripening process (157). Environmental factors and postharvest conditions also play a role in determining the phenolic profile of dates, including caffeic acid content (19). Different extraction methods, such as aqueous and alcoholic extractions, can yield varying levels of phenolic compounds, including caffeic acid. Sukkari dates have shown differences in phenolic content based on the extraction method used (155).

**Table 6:** Fractionation of phenolic and flavonoid compounds.

	**Structure**	**Quantity (mg/100 g DW)**	**References**
**Phenolic acid**			
Caffeic acid	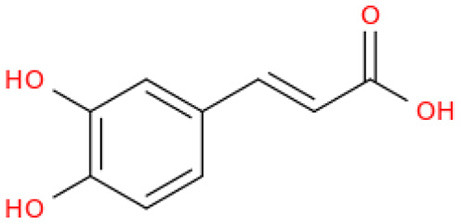	0.019–5.40	(38, 155, 156)
Ferulic acid	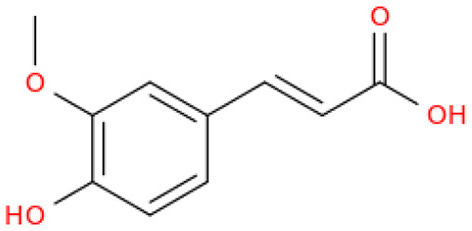	2.01–2.28	(38, 156)
Protocatechuic acid	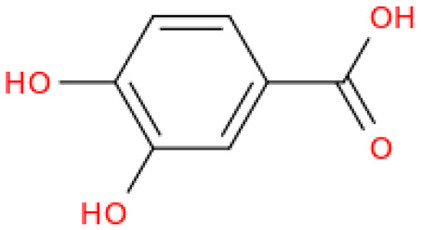	0.893	(38)
Gallic acid	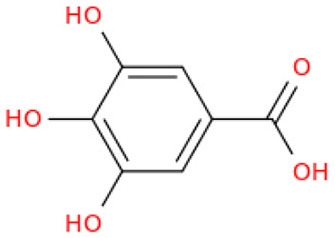	10.24	(38)
p-Coumaric acid	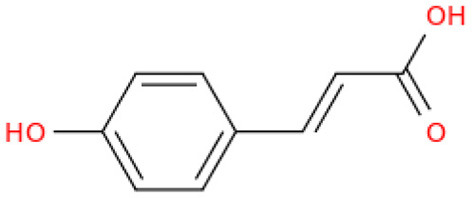	1.37–2.309	(38, 156)
Resorcinol	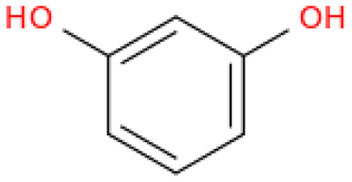	0.022	(38)
Chlorogenic acid	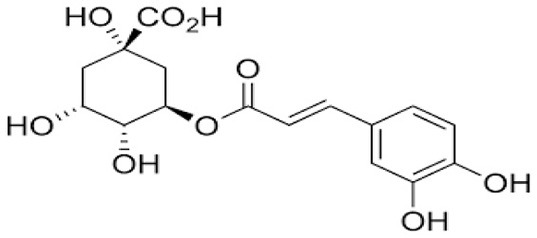	0.135	(38)
Syringic acid	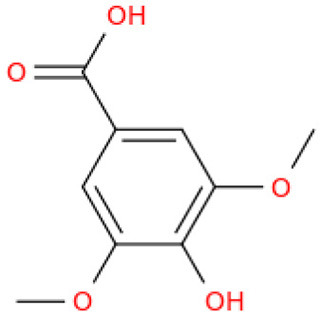	0.60	(38)
**Flavonoids**			
Catechin	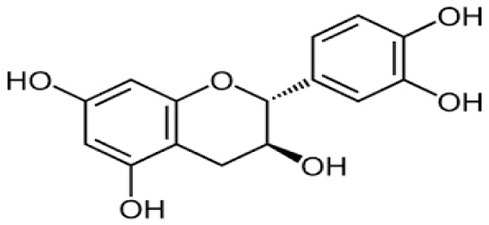	−0.386–7.50	(38, 155)
Quercetin	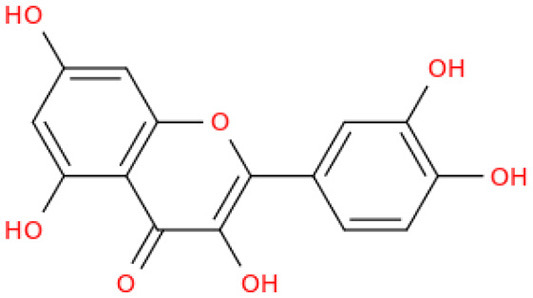	0.838	(38)
Luteolin	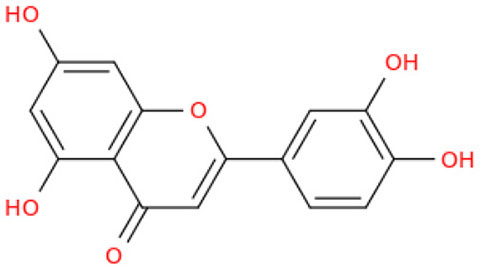	0.028	(38)
Apigenin	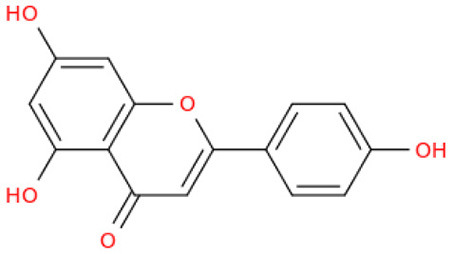	0.181	(38)
Isoquercetrin	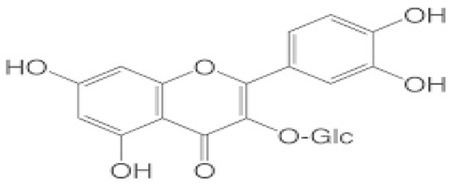	0.271	(38)
Rutin	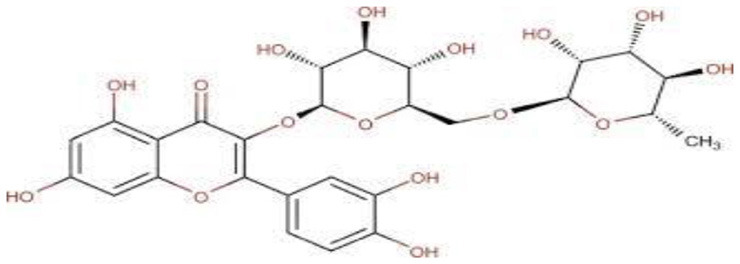	0.665–8.1	(38, 155)

Ferulic acid is a derivative of caffeic acid and is found in various plants, including dates, grains, and vegetables (158). Ferulic acid, a phenolic compound with significant antioxidant properties, is present in Sukkari dates in concentrations ranging from 2.01 to 2.28 mg/100 g dry weight (DW) as reported by 38, and 152. This compound is known for its broad spectrum of therapeutic effects, including anti-inflammatory, antimicrobial, anticancer, and neuroprotective activities, making it a valuable component in both food and pharmaceutical industries (159). The presence of ferulic acid in dates contributes to their nutritional and medicinal value, enhancing their role as a staple food in many cultures (158). Ferulic acid acts as a free radical scavenger and enhances the activity of scavenger enzymes, providing protective effects against oxidative stress (160). It is also used in cosmetics for its photoprotective and skin-brightening properties, although its rapid oxidation can limit its application (160).

The presence of protocatechuic acid (PCA) in Sukkari dates, quantified at 0.893 mg/100 g dry weight (DW) by Hamad et al. (38), highlights the significance of this phenolic compound in dietary sources. PCA is recognized for its diverse pharmacological properties, which contribute to health benefits associated with date consumption. PCA exhibits strong antioxidant activity, which helps combat oxidative stress and may reduce the risk of chronic diseases (161). It has been shown to alleviate inflammation, potentially benefiting conditions like atherosclerosis (258). PCA may inhibit neurodegenerative processes, making it a candidate for therapeutic strategies against diseases such as Alzheimer's (162). Various methods, including reactive extraction, are employed to isolate PCA from plant sources, enhancing its availability for research and application (163). PCA's presence in food products like dates supports its role in promoting health, particularly through its antioxidant and anti-inflammatory properties (13).

The catechin content in Sukkari dates, as reported by Hamad et al. (38) and Saleh et al. (155), ranges from 0.386 to 7.50 mg/100 g dry weight (DW). This range highlights the potential of Sukkari dates as a source of catechins, which are known for their numerous health benefits. Catechins are strong antioxidants that help combat oxidative stress, which is linked to various chronic diseases (259). They exhibit anti-inflammatory properties, potentially reducing the risk of conditions such as cardiovascular diseases (164). Catechins can improve insulin sensitivity and alleviate hyperglycemia, making them beneficial for diabetes management (165).

The presence of gallic acid in Sukkari dates, quantified at 10.24 mg/100 g dry weight (DW) by Hamad et al. (38), highlights its significance as a bioactive compound. Gallic acid, a trihydroxybenzoic acid, is recognized for its antioxidant properties and various health benefits, making it a valuable component in dietary sources. Gallic acid exhibits strong antioxidant activity, effectively scavenging free radicals and reducing oxidative stress, which is crucial in preventing oxidative damage-related diseases (166). *In vitro* studies have demonstrated its ability to inhibit lipid peroxidation and enhance reducing power, showcasing its potential in health promotion (167). Beyond its antioxidant effects, gallic acid possesses anti-inflammatory, antibacterial, and antidiabetic properties, contributing to its therapeutic potential (167). It has been noted for neuroprotective actions, suggesting a role in mitigating neurodegenerative conditions (255). Gallic acid's antibacterial and antifungal properties make it a promising agent for food preservation, potentially extending the shelf life of products like Sukkari dates (168). While the benefits of gallic acid are well-documented, it is essential to consider the variability in its bioavailability and the need for further research to optimize its use in food and therapeutic applications (169).

p-Coumaric acid is found in various plants, including fruits and vegetables, with dates being a significant source (170). The concentration of p-coumaric acid in Sukkari dates, as reported by Hamad et al. (38) and Sheikh et al. (156), ranges from 1.37 to 2.309 mg/100 g dry weight (DW). This phenolic compound is recognized for its various health benefits and is prevalent in many plant-based foods, including dates. p-Coumaric acid exhibits strong free radical scavenging abilities, which can help mitigate oxidative stress in the body (171). Anti-inflammatory Effects: It has been shown to reduce inflammation, making it beneficial for chronic inflammatory conditions (172). This compound possesses antimicrobial properties, contributing to food preservation and health benefits (173). Resorcinol contributes to the antioxidant capacity of dates, which is essential for combating oxidative stress in the body. The presence of resorcinol in Sukkari dates, quantified at 0.022 mg/100 g dry weight (DW) by Hamad et al. (38), highlights the significance of this phenolic compound in the nutritional profile of dates. Resorcinol, a type of dihydroxybenzene, is known for its antioxidant properties and potential health benefits, which can be particularly relevant in the context of date consumption. The low concentration of 0.022 mg/100 g DW suggests that while present, resorcinol is not the predominant phenolic compound in Sukkari dates. The antioxidant properties of resorcinol can enhance the overall health benefits of consuming dates, making them a valuable addition to the diet (174).

Chlorogenic acid (CGA), a polyphenolic compound, is present in various foods, including Sukkari dates, where it has been quantified at 0.135 mg/100 g dry weight (DW) (38). This compound is recognized for its numerous health benefits, particularly in the context of metabolic syndrome and food preservation. CGA exhibits strong antioxidant activity, which helps in preventing oxidative stress-related diseases (175). It plays a role in managing obesity, hypertension, and hyperglycemia by activating AMP-activated protein kinase and inhibiting certain metabolic pathways (176). Anti-inflammatory Effects: CGA has been shown to reduce inflammation, contributing to its potential in treating chronic diseases (177). Due to its antimicrobial properties, CGA is utilized as a natural food additive to enhance food safety and shelf life (113, 114). While the benefits of CGA are well-documented, some studies suggest that its effects can vary based on concentration and the presence of other compounds, indicating a complex interaction within biological systems (175). Syringic acid is recognized for its antioxidant properties, which may help mitigate oxidative stress and inflammation, potentially addressing metabolic risk factors such as hyperglycemia and hypertension (260). Syringic acid, a phenolic compound found in various fruits, including Sukkari dates, has garnered attention for its potential health benefits. According to Hamad et al. (38), the concentration of syringic acid in Sukkari dates is reported to be 0.60 mg/100 g dry weight (DW). This finding aligns with broader research on the nutritional profile of dates, emphasizing their rich content of bioactive compounds. The presence of syringic acid in dates supports their classification as functional foods, contributing to overall health and wellness (178). Quercetin effectively captures reactive oxygen species (ROS) and reduces oxidative stress, which is crucial for preventing cellular damage. Its structure, featuring five hydroxyl groups, enhances its electron-donating ability, making it effective in reducing oxidative stress (179). Quercetin, a prominent flavonoid found in various plant sources, has been identified in Sukkari dates at a concentration of 0.838 mg/100 g dry weight (DW) as reported by Hamad et al. (38). This compound is recognized for its extensive pharmacological properties, including antioxidant, anti-inflammatory, and anticancer effects. The presence of quercetin in Sukkari dates highlights the potential health benefits associated with consuming this fruit, particularly in relation to its bioactive compounds. Quercetin has been linked to various health benefits, including anti-inflammatory effects through the inhibition of pro-inflammatory mediators (180). It has shown potential in cancer prevention by inducing apoptosis and cell cycle arrest, particularly in colorectal cancer (181). Luteolin, a flavonoid found in various fruits and vegetables, has garnered attention for its potential health benefits, particularly in obesity and metabolic diseases. The concentration of luteolin in Sukkari dates, reported as 0.028 mg/100 g dry weight by Hamad et al. (38), indicates a relatively low but significant presence of this compound. This concentration may contribute to the health-promoting properties associated with luteolin, which include anti-inflammatory and antioxidant effects. Luteolin has been shown to inhibit adipocyte differentiation and reduce inflammation in adipose tissue, which can improve metabolic health in obese individuals (182). Luteolin enhances the secretion of beneficial adipokines like adiponectin, which has anti-inflammatory properties. It protects adipocytes from oxidative stress, thereby maintaining their function and promoting metabolic balance (183). Apigenin, a flavonoid found in various fruits and vegetables, has garnered attention for its health benefits, including anti-inflammatory and antioxidant properties. In Sukkari dates, the reported concentration of apigenin is 0.181 mg/100 g dry weight (38). This concentration highlights the potential of Sukkari dates as a dietary source of apigenin, which may contribute to various health-promoting effects. Apigenin exhibits strong antioxidant activity, which helps combat oxidative stress and may reduce the risk of chronic diseases (184). It has been shown to modulate inflammatory pathways, potentially benefiting conditions like cardiovascular diseases and diabetes (29). Epidemiological studies suggest that apigenin may lower the risk of certain cancers, including breast and prostate cancer, by inducing apoptosis in cancer cells (184, 185). The presence of apigenin in Sukkari dates positions them as a valuable addition to diets aimed at enhancing health through natural compounds (186). Apigenin is available as a dietary supplement, which may enhance its bioavailability and therapeutic effects when consumed in higher concentrations (187). Isoquercitrin exhibits strong antioxidant activity, which is crucial in combating oxidative stress linked to various diseases (188). Isoquercitrin, a flavonoid found in Sukkari dates, has been reported at a concentration of 0.271 mg/100 g dry weight (DW) (38). Among various date varieties, Sukkari has been highlighted for its unique phytochemical composition, enhancing its antioxidant potential (13). Sukkari dates are rich in phenolic compounds, including isoquercitrin, which contribute to their health-promoting properties (155). This compound is notable for its antioxidant and potential health benefits, particularly in relation to obesity and liver health. Studies indicate that isoquercitrin can reduce body weight gain and fat accumulation in high-fat diet-induced obesity models. Liver Health: It has been shown to lower hepatic cholesterol and triglyceride levels, suggesting a protective role against non-alcoholic fatty liver disease (NAFLD) (189). Rutin, a flavonoid glycoside derived from quercetin, is a significant bioactive compound found in various plant-based foods, including Sukkari dates. Rutin exhibits a wide range of pharmacological activities, including antioxidant, anti-inflammatory, and anticancer properties. It has been shown to modulate multiple molecular targets involved in carcinogenesis, such as cell cycle mediators and inflammatory cytokines (190, 191). It is used therapeutically as a capillary stabilizing and vasoprotective agent, although no health claims have been approved in the EU (192). Rutin is being explored for its potential use in combination therapies for cancer treatment, as it can enhance the efficacy of other therapeutic agents and reduce drug resistance (191). The concentration of rutin in Sukkari dates, as reported by Hamad et al. (38) and Saleh et al. (155) ranges from 0.665 to 8.1 mg/100 g dry weight (DW). This variability in rutin content can be attributed to several factors, including the plant's metabolic pathways, environmental conditions, and post-harvest processing techniques. Environmental factors and agricultural practices can also influence the rutin content in plant-derived foods (193). The total flavonoid content in Sukkari dates, as reported by Hamad et al. (38), is 1.983 mg/100 g dry weight (DW). This finding aligns with other studies that have explored the flavonoid content in various date cultivars, highlighting the nutritional significance of Sukkari dates. A study by Siddeeg et al. (7) found that the methanolic extract of Sukkari dates contained 3.20 mg CE/100 g, indicating variability in flavonoid content depending on extraction methods. El-Rayes (194) reported higher flavonoid levels in other cultivars, such as Red Sukkary, which had 4.47 mg/100 g fresh weight, suggesting that Sukkari dates may have lower flavonoid concentrations compared to some other varieties (194). The presence of flavonoids in Sukkari dates contributes to their antioxidant properties, which are beneficial for health (7). The nutritional profile of Sukkari dates, including their flavonoid content, supports their potential as a functional food (124).

**Figure 2:**
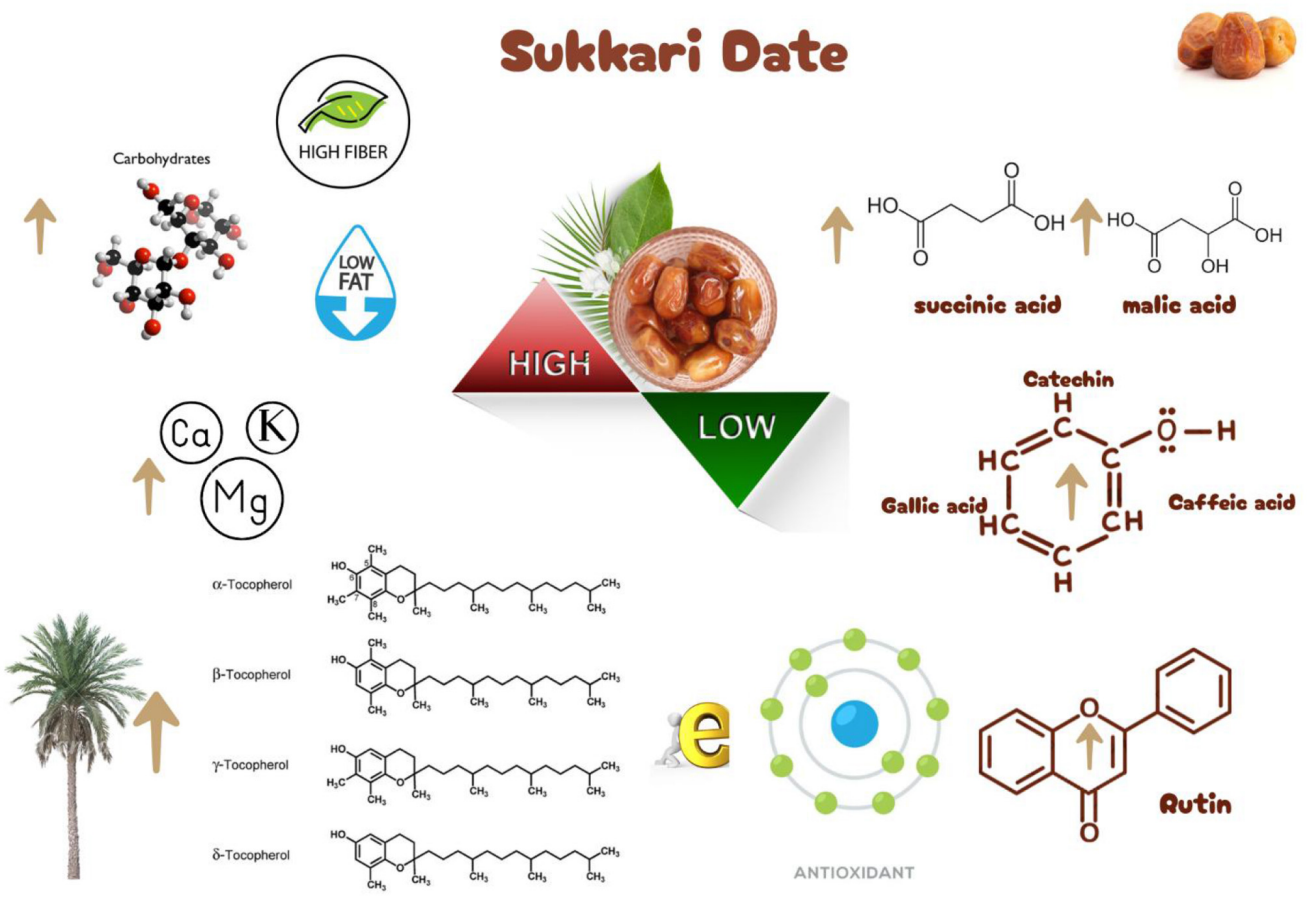
Nutritional profile, minerals, and beneficial phytochemicals of Sukkari dates.

The results presented reveal that Sukkari dates are recognized for their exceptional nutritional profile, characterized by high levels of essential minerals, dietary fiber, carbohydrates, and beneficial phytochemicals. These attributes contribute significantly to their health benefits and overall nutritional value. The following sections detail the key components of Sukkari dates (Figure 2). Sukkari dates are rich in minerals, particularly calcium, potassium, and magnesium, which are vital for various bodily functions (7, 35). Potassium levels can reach up to 9,619 mg/kg, supporting cardiovascular health and potentially aiding in hypertension management (95). Sukkari dates contain approximately 78.32% sugars, primarily glucose and fructose, alongside 3.15% dietary fiber, enhancing digestive health (7). They also exhibit a notable protein content of 5.27% and low-fat levels, making them a balanced energy source (37). Sukkari dates are abundant in tocopherols, phenolic compounds, and flavonoids, which provide antioxidant properties that combat oxidative stress (6, 7). Specific acids such as succinic and malic acids, along with gallic acid, caffeic acid, and rutin, contribute to their health-promoting effects (6). While Sukkari dates offer numerous health benefits, it is essential to consider that their high sugar content may not be suitable for individuals with certain metabolic conditions, such as diabetes. Balancing their consumption with overall dietary needs is crucial for optimal health outcomes.

## Vitamins of Sukkari dates

The concentration of vitamin B1 (thiamin) in Sukkari dates is reported to be 0.55 mg/g dry weight, highlighting its nutritional significance (47). Thiamin plays a crucial role in carbohydrate metabolism and is essential for maintaining healthy cardiac and neural functions. Thiamin is vital for the production of ATP, ribose, NAD, and DNA, serving as a cofactor for key enzymes in metabolic pathways (195). Deficiency can lead to severe health issues, including beriberi and Wernicke-Korsakoff syndrome, particularly in populations with limited dietary diversity (195, 261). The presence of vitamin B3 (niacin) in Sukkari dates, quantified at 0.40 mg/g dry weight, highlights its nutritional significance, while the absence of vitamin B2 (riboflavin) indicates a specific deficiency in this cultivar (47). Niacin plays a vital role in the formation of NAD and NADP, crucial for redox reactions in metabolic processes. It is essential for energy metabolism and various cellular functions (196). While Sukkari dates are rich in vitamin B3, the lack of vitamin B2 may limit their effectiveness as a comprehensive source of B vitamins. This highlights the need for dietary diversity to ensure adequate intake of all essential nutrients. The analysis of vitamins B6 and B5 in Sukkari dates reveals significant findings regarding their nutritional content. Sukkari dates contain a notable concentration of vitamin B6 at 2.38 mg/g dry weight, while vitamin B5 was not detected in these dates (47). This highlights the potential of Sukkari dates as a source of vitamin B6, which is essential for various metabolic processes. Vitamin B6 plays a crucial role in amino acid metabolism, neurotransmitter synthesis, and immune function (19). Vitamin B5 was not detected in Sukkari dates, indicating a lack of this nutrient in this specific variety (197). Folate is vital for nucleic acid biosynthesis and amino acid metabolism, which are essential for cell division and growth (198). Adequate folate levels are linked to reduced risks of neural tube defects during pregnancy and may prevent megaloblastic anemia (198, 199). The vitamin B9 content in Sukkari dates is reported to be 0.05 mg/g dry weight, indicating a modest presence of this essential nutrient. Vitamin B9, or folate, is crucial for various biological functions, including DNA synthesis and repair, and plays a significant role in cellular metabolism and immune function (47). Dates, including Sukkari, are recognized for their rich nutritional profile, containing various vitamins and minerals, with vitamin B9 being one of them (10, 35). The presence of folate in dates contributes to their classification as functional foods, beneficial for overall health and wellness (10).

The presence of vitamin B12 in Sukkari dates, quantified at 0.55 mg/g dry weight, highlights the nutritional significance of this fruit (47). The Sukkari date variety has been reported to contain a significant amount of vitamin E, quantified at 19.74 mg/g dry weight, while vitamin A was not detected in these dates (47). This finding highlights the nutritional profile of Sukkari dates, particularly their antioxidant properties attributed to vitamin E. Sukkari dates exhibit a high concentration of vitamin E, specifically α-tocopherol, which is known for its antioxidant properties (133). The presence of vitamin E in dates contributes to their health benefits, including anti-inflammatory effects and potential nephroprotective properties (133). Despite the high vitamin E content, Sukkari dates lack detectable levels of vitamin A, which is essential for vision and immune function (200).

## Therapeutic effects of Sukkari dates

Sukkari dates (*Phoenix dactylifera* L.) exhibit a range of therapeutic effects, including hepatoprotective, antibacterial, and metabolic benefits. These effects are attributed to their rich phytochemical composition, which includes phenolic compounds and antioxidants. Sukkari dates have been shown to improve glycemic control, enhance lipid profiles, and support weight loss, making them beneficial for managing conditions like diabetes and obesity. Additionally, they have been found to enhance memory functions and brain cholinergic transmission, further highlighting their potential in cognitive health. The following sections detail these therapeutic effects. Sukkari dates exhibit various therapeutic effects, particularly in managing metabolic disorders and enhancing liver health. Research indicates that Sukkari date extracts can significantly reduce liver enzyme levels, improve cognitive function in diabetic models, and aid in weight management and metabolic health (Table 7). The study by Kadir et al. (201) highlights the hepatoprotective effects of Sukkari date extract in male Wistar rats subjected to liver damage via meloxicam. The results indicate that the extract significantly reduced liver enzyme levels, suggesting its potential in mitigating non-steroidal anti-inflammatory drug (NSAID)-induced liver toxicity. Sukkari date extract may protect the liver by neutralizing free radicals, which are implicated in oxidative stress and liver damage (201). The extract notably decreased ALT and AST levels, with values dropping to 67.17 U/L and 63.00 U/L for the 500 mg/kg and 1,000 mg/kg doses, respectively (201). Similar studies have shown that extracts from dates can ameliorate liver damage induced by carbon tetrachloride and thioacetamide, further supporting the hepatoprotective properties of date extracts (202, 203). The findings suggest that natural extracts like Sukkari dates could serve as alternative treatments for liver toxicity, especially in regions where such resources are abundant (201, 262). The findings suggest that Sukkari date extract could be a natural alternative for liver protection in clinical settings, especially for patients requiring NSAID treatment (201).

**Table 7:** Therapeutic effects of Sukkari date.

**Source**		**Objective**	**Study design/duration of treatment**	**Results**
1	Kadir et al. (201)	The study investigated the hepatoprotective effects of Sukkari date extract on male Wistar rats that were induced with liver damage using meloxicam.	The experiment involved 24 male Wistar rats divided into four groups, each containing six rats. Positive control group (K2): Received 30 mg/kg body weight (BW) of meloxicam and 1% Na CMC. Treatment group 1 (P1): Received 500 mg/kg BW of Sukkari date extract along with 30 mg/kg BW of meloxicam. Treatment group 2 (P2): Received 1,000 mg/kg BW of Sukkari date extract along with 30 mg/kg BW of meloxicam. Negative control group (K1): Received only 1% Na CMC without meloxicam. The study lasted for 12 days, during which the rats were monitored for changes in liver enzyme levels.	The findings suggest that Sukkari date extract significantly reduced the levels of ALT (Alanine aminotransferase) ALT levels to 67.17 U/L and 63.00 U/L for the 500 and 1,000 mg/kg doses, respectively. Similarly, AST (Aspartate aminotransferase) levels decreased to 142.83 and 125.83 U/L in these groups in male Wistar rats that were induced with a toxic dose of meloxicam. This suggests that Sukkari date extract may help in mitigating liver toxicity associated with NSAID use, likely due to its ability to neutralize free radicals.
2	Anindita et al. (204)	The study evaluated the antibacterial effectiveness of methanol extract from the Sukkari variety of dates against three bacteria known to cause acne: *Propionibacterium acnes, Staphylococcus epidermidis*, and *Staphylococcus aureus*.	The research involved testing various concentrations of the date extract (50%, 60%, 70%, 80%, and 90%) using the Kirby–Bauer method, which measures the zone of inhibition around antibiotic disks to determine bacterial sensitivity.	The study found that the methanol extract from the Sukkari variety of dates did not effectively inhibit the growth of acne-causing bacteria, specifically *Propionibacterium acnes, Staphylococcus epidermidis*, and *Staphylococcus aureus*. This indicates that Sukkari dates are not suitable for development into anti-acne skin care products. The study highlighted that the Sukkari dates used were in a very ripe stage, which may have contributed to their lower antibacterial effectiveness. As dates ripen, they tend to accumulate more sugars and fewer secondary metabolites, which are often responsible for antibacterial activity.
3	Aljutaily et al. (11)	The effects of intermittent fasting, fermented camel milk and 10% Sukkari date (FCM-D) on weight loss, blood profile, and antioxidant.	The study utilized Wistar rats, specifically 48 rats weighing between 125 and 140 g. The rats were fed a high-fat diet for 45 days to induce obesity. This diet was modified to include camel hump fat instead of lard, ensuring a consistent high-fat intake. Experimental Groups: After the obesity induction, the rats were divided into six groups, each receiving different treatments: Control group (no treatment). Intermittent fasting (IF) group, fasting for 16 hours daily. Group receiving fermented camel milk (FCM). Group receiving FCM with IF. Group receiving fermented camel milk incorporating 10% Sukkari date (FCM-D). Group receiving FCM-D with IF. After 6 weeks of treatment, blood samples were analyzed for various parameters.	**Weight Loss:** The study found that intermittent fasting (IF) combined with fermented camel milk incorporating Sukkari date (FCM-D) led to significant weight loss in obese rats. **Blood Glucose Levels**: The lowest blood glucose levels were recorded in rats that underwent IF along with FCM or FCM-D. Lipid Profile Improvement: The combination of IF with FCM or FCM-D led to reductions of 40% for TG, 37% for CHO, 66% for LDL-c, and 40% for VLDL-c were noted, along with a 34% increase in high-density lipoprotein (HDL-C) levels. **Atherogenic Index (AI**): Combining IF with FCM or FCM-D reduced AI by 42% and 59%, respectively. **Leptin and Adiponectin Levels**: The treatments also affected hormone levels related to fat storage and metabolism. Leptin levels, which are typically high in obesity, decreased significantly with the treatments. The most effective reductions were seen with the combination of FCM-D and IF, which resulted in a 20.38% decrease in leptin levels. Conversely, adiponectin levels, which are beneficial for metabolic health, increased significantly with the treatments. **Oxidative Stress Markers:** The study found that the levels of oxidative stress markers, such as malondialdehyde (MDA), were significantly reduced in rats treated with FCM-D and IF. This indicates an improvement in antioxidant status.
4	Aljutaily et al. (18)	The research highlights the potential of using date fiber, a by-product of date fruit processing, as a functional food ingredient. This not only addresses obesity but also valorizes agricultural waste, contributing to environmental sustainability.	The forty-eight male albino rats were divided into six groups, including a negative control group (non-obese) and five obese groups. The obese groups were further divided to receive different treatments: one group received Orlistat (a medication for weight loss), while the others were fed biscuits supplemented with varying levels of date fiber (DF) at 5%, 10%, and 15%.	The study demonstrated that biscuits supplemented with date fiber (DF) at different levels (5%, 10%, and 15%) effectively helped control body weight in obese rats. The most significant weight loss was observed in the group that received the highest DF supplementation (15%).
5	Elbably et al. (209)	The study investigated the effects of Sukkari date extract on diabetes induced by streptozotocin (STZ) in male rats.	The study involved 40 male albino rats divided into two main groups: Control Group (Group I): 10 rats that served as normal controls. STZ Group (Group II): 30 rats injected with STZ to induce diabetes, further divided into three subgroups after 48 h: Subgroup a: Diabetic rats not treated (diabetic control). Subgroup b: Diabetic rats treated with aqueous Sukkari date extract (10 ml/day orally for 60 days). Subgroup c: Diabetic rats treated with Amaryl tablets (1 ml/rat/day orally for 60 days). After 60 days of treatment, blood samples were analyzed for various parameters.	The results indicated that the effects of Sukkari date extract were comparable to those of Amaryl (a known antidiabetic medication). Both treatments led to significant improvements in the measured parameters, suggesting that Sukkari date extract could be a viable alternative or complementary therapy for diabetes, as evidenced by the significant reduction in malondialdehyde (MDA) levels and an increase in superoxide dismutase (SOD) levels. This suggests that the extract may help mitigate oxidative stress associated with diabetes.
6	Anshar et al. (212)	The study on the nephroprotective effect of Sukkari date extract involved a systematic experimental design.	A total of 24 male white Wistar rats were used in the experiment. These rats were divided into four treatment groups to evaluate the effects of different interventions. Treatment Groups: Negative Control Group (K1): This group received 1% NaCMC, serving as a baseline for comparison. Positive Control Group (K2): This group was administered 30 mg/kg body weight (BW) of meloxicam, which is known to induce kidney damage. Treatment Group 1 (P1): This group received 2 ml of Sukkari date extract at a dose of 500 mg/kg BW. Treatment Group 2 (P2): This group received 2 ml of Sukkari date extract at a higher dose of 1,000 mg/kg BW. The rats were treated with Sukkari date extract for 11 days. On the 12th day, all groups except the negative control were induced with meloxicam to assess the protective effects of the extract.	Sukkari date extract (*Phoenix dactylifera*) has a significant nephroprotective effect against kidney damage induced by meloxicam in rats. The extract was able to maintain blood urea nitrogen (BUN) levels within normal ranges despite exposure to toxic doses of meloxicam, indicating its protective properties for kidney health. The findings suggest that the antioxidant properties of Sukkari dates, attributed to their content of flavonoids, phenolics, and vitamins, play a crucial role in mitigating oxidative stress and preventing kidney damage caused by drug toxicity.
7	Sheikh et al. (156)	Comparative study of neuropharmacological, analgesic properties of Ajwah, Safawy and Sukkari cultivars of date palm (*Phoenix dactylifera*).	Acute Toxicity Test: Mice were divided into groups and treated with different doses of date palm extract. They were observed for 72 h to record any signs of toxicity or mortality. Mice were divided into groups and treated with different doses of date palm extract. They were observed for 72 h to record any signs of toxicity or mortality. **Neuropharmacological Tests:** Pentobarbitone-induced Sleeping Time Test: Mice were treated with date extracts and then given pentobarbitone to induce sleep. The onset and duration of sleep were recorded. **Open Field Test**: Mice were placed in an open field, and their movements were tracked to assess locomotor activity Hole Board Test: The number of head dips by the mice in a board with holes was recorded to evaluate exploratory behavior **Antinociceptive Tests:** Acetic Acid Induced Writhing Test: Mice were given acetic acid, and the number of writhes was counted to measure pain response **Hot Plate Test:** Mice were placed on a hot plate, and the time taken to lick their paws or jump was recorded to assess pain sensitivity.	The findings these extracts could be beneficial for mild relaxation and pain relief. However, the effects are not as strong as those produced by conventional medications, indicating that while date palm extracts may be useful, they should not replace standard treatments for serious conditions. The presence of important bioactive compounds such as trans-ferulic acid, (+)-catechin, and (–)-epicatechin in the date extracts is likely responsible for the observed neuroprotective and analgesic activities. These compounds are known for their antioxidant properties, which can help protect brain cells and reduce inflammation.
8	Abdelbaky et al. (13)	The study on the antioxidant and anticancer assessment of three varieties of date fruits employed several methods to evaluate the phytochemical profiles and biological activities of the extracts.	The antioxidant activity of the fruit extracts was evaluated using the DPPH· (2,2-diphenyl-1-picrylhydrazyl) method. The cytotoxic effects of the extracts (ethyl acetate, hydroethanol, hydromethanol, and aqueous solutions) on human cancer cell lines were assessed using the Sulforhodamine B (SRB) assay.	**Antioxidant Activity:** The ethyl acetate extract of Sukkari date fruits demonstrated the highest antioxidant potential, with an IC50 value of 132.4 ± 0.3 μg·mL^−1^. This indicates that it was effective at lower concentrations in scavenging free radicals. **Cytotoxicity Against Cancer Cell Lines**: The methanol extract of Sukkari fruits also showed significant anticancer activity, with an IC50 value of 119 ± 3.5 μg·mL^−1^ against the same breast cancer cell line.
9	Famuyiwa et al. (109)	The study uses specific methods to compare the glycemic and insulin responses to dates and oral dextrose in both diabetic and non-diabetic subjects.	The study involved 16 well-controlled non-insulin-dependent diabetic patients and 10 healthy subjects. This diverse group allowed for a comparison between different metabolic responses to the tested substances. **Oral Glucose Tolerance Test (OGTT)**: An OGTT was conducted using 75 grams of dextrose. **Oral Dates Tolerance Test (ODTT):** ODTT was performed using an isocaloric amount of using semi-dried Sukkari dates (Al-Qassim) in the ripe (tamer) stage, specifically 110 g, which equated to 300 kcal. **Measurement of Glucose Levels:** Blood glucose levels were measured at various time intervals (60–120 min) after the ingestion of dextrose and dates. **Measurement of Glucose Levels:** Blood glucose levels were measured at various time intervals (60–120 min) after the ingestion of dextrose and dates.	**Higher Glucose Levels with Dextrose**: In the diabetic patients, the glucose levels were significantly higher after consuming dextrose compared to dates. This was observed at various time points, specifically from 60 to 120 min post-ingestion. The results indicated that dextrose led to a more pronounced increase in blood glucose levels than dates did. **Incremental Area Under Curve (IAUC):** The study calculated the incremental area under the curve (IAUC) for glucose over a 2-h period. The IAUC for glucose following dextrose was significantly larger, measuring −76.1 ± 5.2 mmol/liter, compared to the IAUC for dates, which was −51.4 ± 3.1 mmol/liter. This difference was statistically significant (*p* < 0.05), highlighting those dates resulted in a lower glycemic response than dextrose. **Comparison Between Diabetic and Non-Diabetic Subjects**: While the specific results for non-diabetic subjects were not detailed in the provided context, the overall trend suggests that dates may be a better option for managing blood sugar levels in both diabetic and non-diabetic individuals due to their lower impact on glucose levels. The findings suggest that incorporating dates into the diet may be beneficial for individuals looking to manage their blood sugar levels, particularly for those with diabetes. The lower glycemic response associated with dates compared to dextrose indicates that dates could be a healthier alternative for sweetening foods or as a snack.
10	Alolyan et al. (220)	The study employed several methods to investigate the postprandial antioxidative response to the ingestion of formulated date-based bars (DBBs) and fruit-based bars (FBBs) in healthy individuals.	The study involved twenty healthy participants with an average age of 21.4 years. Each participant ingested 140 g (510 Kcal) of either type of bar. Blood samples were taken from participants at various time intervals after ingestion (0, 60, 120, and 180 min). The total phenolic content (TPC), total antioxidant capacity (T-AOC), malondialdehyde (MDA), and superoxide dismutase (SOD) levels in the plasma were analyzed.	The findings indicate that DBBs significantly enhance postprandial total phenolic content (TPC) and total antioxidant capacity (T-AOC) compared to FBBs. The TPC peaked at 120 min after ingestion of DBBs, suggesting that these bars are effective in increasing beneficial phenolic compounds in the bloodstream. A notable decrease in malondialdehyde (MDA) levels was observed 180 min after consuming DBBs, indicating a reduction in oxidative stress. In contrast, FBBs did not show a significant change in MDA levels, highlighting the superior antioxidative properties of DBBs. The study found a significant increase in superoxide dismutase (SOD) levels 120 min after the ingestion of DBBs, while FBBs did not elicit a considerable response. This suggests that DBBs may enhance the body's enzymatic antioxidant defenses more effectively than FBBs.
11	Balakrishnan et al. (221)	The study employed several methods to evaluate the effects of Saudi Arabian date fruit varieties on Freund's complete adjuvant (FCA)-induced arthritis in rats.	The research utilized rats as the animal model for inducing rheumatoid arthritis through Freund's complete adjuvant (FCA) injection, The rats were treated orally for 30 days with methanolic extracts from four different varieties of Saudi Arabian date fruits: Sukkari, Ekhlass Almajmaah, Abu Minifee, and Dawee. The dosage for these extracts was 500 mg/kg body weight (BW). A standard treatment comparison was made using Indomethacin, administered at a dosage of 10 mg/kg BW. **The study assessed several parameters to measure the effects of the treatments:** ^*^The severity of arthritis was evaluated by measuring paw volume and calculating an arthritis score. ^*^The body weight of the rats was monitored throughout the study to assess any changes due to the treatments. ^*^Levels of renal markers and bone metabolic enzymes were measured to evaluate the physiological effects of the treatments on kidney function and bone health. ^*^The antioxidant activities of the date fruit extracts were also assessed, which is crucial for understanding their potential protective effects against oxidative stress associated with arthritis.	^*^All varieties of date fruit extracts (Sukkari, Ekhlass Almajmaah, Abu Minifee, and Dawee) significantly reduced the symptoms of arthritis, indicating a reduction in inflammation and joint swelling. ^*^The treatment with date fruit extracts helped maintain the body weight of the rats throughout the study period. This suggests that the extracts may have a protective effect against the weight loss often associated with inflammatory conditions like arthritis. ^*^The study found that the date fruit extracts positively influenced renal marker levels and bone metabolic enzymes. This indicates that the extracts may help in preserving kidney function and bone health, which can be compromised in arthritic conditions. ^*^The extracts may help combat oxidative stress, which is often elevated in inflammatory diseases like arthritis.
12	Dewi (224)	The study on the antioxidant effects of ethanol extract from Sukkari dates (*Phoenix dactylifera*) on male rats induced with paracetamol.	The study utilized 20 male Wistar rats, which were divided into four treatment groups. Group 1: This group served as the negative control and was administered paracetamol at a dose of 2.5 g/Kg body weight (KgBB). Groups II–IV: These groups received the ethanol extract of Sukkari dates for 10 days at varying doses of 250 mg/KgBB, 500 mg/KgBB, and 1,000 mg/KgBB, respectively. This setup was designed to assess the dose-dependent effects of the extract on antioxidant activity. On the 7th day of the experiment, all groups except the control were induced with paracetamol to create a state of oxidative stress. Blood samples were collected from the rats on days 8, 9, and 10 of the study. This sampling was essential for measuring the levels of malondialdehyde (M).	The results indicated that the doses of 250 mg/KgBB and 1,000 mg/KgBB of the date extract significantly lowered MDA levels on the 10th day of treatment. This finding implies that the antioxidant effect of the extract may vary with dosage. The findings suggest that regular consumption of Sukkari dates could contribute to reducing oxidative stress and improving overall health, Given the presence of flavonoids, phenolic compounds, and vitamins (C, A, E, and β-carotene) in Sukkari dates.
13	Algeffari et al. (100)	The Study Glycemic Indices for 17 varieties of dates grown in Saudi Arabia.	The study focuses on seventeen different varieties of dates that are cultivated in Saudi Arabia. **Measurement of Glycemic Index (GI):** The glycemic index of each date variety is determined through a controlled study involving human participants. This involves administering a specific amount of each date variety to the participants and measuring their blood glucose levels at regular intervals after consumption. **Calculation of Glycemic Load (GL**): Alongside the glycemic index, the study calculates the glycemic load for each date variety. Glycemic load takes into account both the glycemic index and the carbohydrate content of the food.	The mean (SEM) GI of the date samples was 55.2 (7.7) (range, 42.8–74.6). Sellaj and Maktoomi exhibited the highest GI whereas Shaqra, Sukkary, and Sag'ai had the lowest GI [42.8 [5.5], 43.4 [4.7], and 44.6 [6]], respectively.

However, further research is necessary to confirm these effects in human subjects and to explore the long-term safety and efficacy of date extracts. While the results are promising, it is essential to consider that the protective effects observed in animal models may not fully translate to humans, necessitating cautious interpretation and further investigation. The study by Anindita et al. (204) evaluated the antibacterial effectiveness of methanol extract from Sukkari dates against acne-causing bacteria. The study tested various concentrations (50%−90%) of the methanol extract using the Kirby–Bauer method. Results indicated no significant inhibition of Propionibacterium acnes, Staphylococcus epidermidis, and Staphylococcus aureus, with all bacteria showing resistance (204). The ripe stage of the dates may have contributed to the low antibacterial activity, as ripening increases sugar content and decreases secondary metabolites responsible for antibacterial properties (204). While Sukkari dates did not exhibit antibacterial properties, other natural extracts have shown promise, indicating that further exploration of different plant sources may yield effective anti-acne treatments (205).

The study by Aljutaily et al. (11) highlights the beneficial effects of intermittent fasting (IF) combined with fermented camel milk incorporating Sukkari date (FCM-D) on metabolic health in obese rats. This combination resulted in significant weight loss, improved blood glucose levels, and enhanced lipid profiles. The findings suggest that such dietary interventions could be effective strategies for managing obesity and related metabolic disorders. The lowest blood glucose levels were observed in rats receiving IF with FCM or FCM-D, indicating improved glycemic control (11, 18). Leptin levels decreased significantly, particularly with FCM-D and IF, while adiponectin levels increased, promoting metabolic health. Oxidative stress markers, such as malondialdehyde (MDA), were significantly reduced, indicating improved antioxidant status (11, 18). The research by Aljutaily et al. (18) highlights the potential of using date fiber (DF), a by-product of date fruit processing, as a functional food ingredient to address obesity and valorize agricultural waste. The study demonstrated that biscuits supplemented with varying levels of DF (5%, 10%, and 15%) effectively helped control body weight in obese rats, with the most significant weight loss observed in the group receiving 15% DF supplementation. This approach not only contributes to obesity management but also promotes environmental sustainability by utilizing agricultural by-products. The study found that DF supplementation in biscuits led to significant weight control in obese rats, with the highest DF level (15%) showing the most substantial weight loss (11, 18). DF supplementation also improved lipid profiles, reducing total cholesterol and triglycerides, which are critical factors in managing obesity-related health issues (11, 18). Date fibers are rich in magnesium and potassium, contributing to their nutritional value when used in food products (206). The incorporation of DF in biscuits enhances their antioxidant properties and total phenolic content, which are beneficial for health (206, 207). In this regard, utilizing date fiber valorizes agricultural waste, reducing environmental impact and promoting sustainability (208). While the study shows promising results in using date fiber to combat obesity, it is essential to consider consumer acceptance and sensory attributes. Research indicates that biscuits with up to 10% DF are well-accepted by consumers, balancing health benefits with taste and texture (206). Further studies could explore the long-term effects of DF consumption in humans and its potential market applications. The investigation into the effects of Sukkari date extract on diabetes induced by streptozotocin (STZ) reveals promising results, indicating its potential as an alternative or complementary therapy to conventional antidiabetic medications like Amaryl. The extract demonstrated significant improvements in various biochemical parameters, including a reduction in malondialdehyde (MDA) levels and an increase in superoxide dismutase (SOD) levels, suggesting its role in mitigating oxidative stress associated with diabetes (209). Sukkari date extract significantly reduced blood glucose levels in STZ-induced diabetic rats, comparable to Amaryl (209). The date seed extract also improved lipid profiles, decreasing cholesterol and triglyceride levels (210). Beyond metabolic improvements, Sukkari date seed extract has been shown to alleviate cognitive impairments in diabetic models, enhancing memory functions and brain cholinergic transmission (211). While the findings support the therapeutic potential of Sukkari date extract, further research is necessary to fully understand its mechanisms and long-term effects in human subjects. Additionally, the reliance on traditional remedies may not always align with evidence-based practices, necessitating cautious integration into clinical settings. The nephroprotective effects of Sukkari date extract (*Phoenix dactylifera*) against meloxicam-induced kidney damage have been substantiated through systematic experimental studies. The extract demonstrated significant protective properties, maintaining blood urea nitrogen (BUN) levels within normal ranges despite exposure to toxic doses of meloxicam. This effect is attributed to the antioxidant properties of Sukkari dates, which are rich in flavonoids, phenolics, and vitamins, crucial for mitigating oxidative stress and preventing kidney damage (212). The flavonoids and phenolics in Sukkari dates help reduce oxidative stress, a key factor in nephrotoxicity (212). In the study, treatment groups receiving Sukkari date extract showed BUN levels of 20.48 mg/dl and 24.86 mg/dl, significantly lower than the positive control group at 41.55 mg/dl (212). Other studies have shown that date extracts also protect against nephrotoxicity induced by gentamicin, highlighting the broader applicability of date extracts in renal protection (213, 214). Similar findings were observed with Ajwa dates, which also demonstrated nephroprotective effects against meloxicam toxicity (215). The study by Sheikh et al. (156) highlights the neuropharmacological and analgesic properties of three date palm cultivars: Ajwah, Safawy, and Sukkari. The findings suggest that these extracts can provide mild relaxation and pain relief, although they are not as potent as conventional medications. The presence of bioactive compounds such as trans-ferulic acid, (+)-catechin, and (–)-epicatechin is likely responsible for these effects, as they exhibit antioxidant properties that protect brain cells and reduce inflammation. Trans-ferulic acid, (+)-catechin, and (–)-epicatechin were identified as key compounds. These compounds are known for their neuroprotective and anti-inflammatory activities (127, 156). While date palm extracts show promise for mild conditions, they should not replace conventional treatments for serious ailments, as their effects are comparatively limited (156). The study by Abdelbaky et al. (13) highlights the significant antioxidant and anticancer properties of date fruits, particularly focusing on the Sukkari variety. The ethyl acetate extract of Sukkari dates exhibited the highest antioxidant activity, with an IC50 value of 132.4 ± 0.3 μg·mL^−1^, indicating its effectiveness in scavenging free radicals. Additionally, the methanol extract of Sukkari dates demonstrated notable cytotoxicity against breast cancer cells, with an IC50 of 119 ± 3.5 μg·mL^−1^. The ethyl acetate extract of Sukkari dates showed superior antioxidant potential. Comparative studies indicate that other date varieties also possess significant antioxidant properties, with varying IC50 values (13, 139). The methanol extract of Sukkari dates was effective against MDA-MB-231 breast cancer cells. Other varieties, such as Siwi, also demonstrated promising anticancer activities, with IC50 values as low as 99 ± 1.6 μg·mL^−1^ (13, 216). The research conducted by Famuyiwa et al. (109) highlights the significant differences in glycemic responses between dextrose and dates in diabetic patients. The study found that dextrose consumption resulted in markedly higher blood glucose levels compared to dates, particularly noted at 60–120 min post-ingestion. The incremental area under the curve (IAUC) for glucose was significantly greater for dextrose, indicating a more pronounced glycemic response. Dextrose led to an IAUC of −76.1 ± 5.2 mmol/liter, while dates had an IAUC of −51.4 ± 3.1 mmol/liter, with a statistically significant difference (*p* < 0.05) (109). Dates may be a preferable option for managing blood sugar levels due to their lower glycemic impact, suggesting they could be beneficial for both diabetic and non-diabetic individuals (109, 217). The findings advocate for the inclusion of dates in diets aimed at blood sugar management, contrasting with the common advice to avoid them (217). Conversely, while dates show a lower glycemic response, some studies suggest that individual responses to carbohydrates can vary significantly, indicating that personal dietary management should consider these differences (218, 219).

The study by Alolyan et al. (220) highlights the superior postprandial antioxidative response of date-based bars (DBBs) compared to fruit-based bars (FBBs) in healthy individuals. The research indicates that DBBs significantly enhance total phenolic content (TPC) and total antioxidant capacity (T-AOC) post-ingestion, with TPC peaking at 120 min. This suggests that DBBs are effective in increasing beneficial phenolic compounds in the bloodstream. Additionally, a notable decrease in malondialdehyde (MDA) levels was observed 180 min after consuming DBBs, indicating a reduction in oxidative stress, a response not seen with FBBs. Furthermore, DBBs significantly increased superoxide dismutase (SOD) levels, enhancing enzymatic antioxidant defenses more effectively than FBBs. DBBs contain higher bound phenolic content compared to FBBs, contributing to their superior antioxidative properties (220). The nutritional composition of DBBs includes higher levels of certain minerals and amino acids, which may contribute to their health benefits (16). While the study emphasizes the superior antioxidative properties of DBBs, it is important to consider the broader context of dietary choices. FBBs, despite their lower postprandial antioxidative response, still offer nutritional benefits, such as higher free phenolic content and different amino acid profiles, which may be advantageous in other health contexts (16). Further research could explore the long-term health impacts of these bars and their role in a balanced diet. The study by Balakrishnan et al. (221) highlights the therapeutic potential of various Saudi Arabian date fruit extracts in alleviating symptoms of Freund's complete adjuvant (FCA)-induced arthritis in rats. The findings suggest that these extracts not only reduce inflammation and joint swelling but also help maintain body weight, indicating a protective effect against weight loss associated with arthritis. Furthermore, the extracts positively influence renal markers and bone metabolic enzymes, suggesting benefits for kidney function and bone health, while also combating oxidative stress prevalent in inflammatory conditions. All date fruit extracts (Sukkari, Ekhlass Almajmaah, Abu Minifee, Dawee) significantly reduced paw volume and arthritis scores, indicating effective anti-inflammatory properties (221). Similar findings were reported for date palm seed extracts, which also lowered inflammatory cytokines and improved overall health markers in arthritic rats (222). Date fruit extracts helped maintain body weight throughout the study, contrasting with the typical weight loss seen in arthritic conditions (221). This aligns with findings from other studies where herbal treatments preserved body weight in arthritic models (223). Positive effects on renal markers and bone metabolic enzymes were observed, suggesting that date fruit extracts may protect kidney function and bone integrity in arthritic conditions (221). While the results are promising, it is essential to consider that the efficacy of herbal treatments can vary based on individual responses and the complexity of arthritis, necessitating further research to establish standardized treatment protocols. The antioxidant effects of Sukkari dates (*Phoenix dactylifera*) have been demonstrated in various studies, particularly in mitigating paracetamol-induced oxidative stress in male rats. The findings indicate that both 250 mg/kg and 1,000 mg/kg doses of the date extract significantly reduced malondialdehyde (MDA) levels, suggesting a dose-dependent antioxidant effect. This is supported by the presence of bioactive compounds such as flavonoids, phenolic compounds, and vitamins, which contribute to the overall health benefits of Sukkari dates (224). Sukkari dates contain flavonoids, phenolics, and vitamins (C, A, E, and β-carotene) that enhance antioxidant activity (225). Significant decreases in MDA levels were observed, indicating reduced lipid peroxidation and oxidative stress (226). Studies show that date extracts can reverse paracetamol-induced liver damage by improving enzyme levels (ALT, AST) and reducing bilirubin concentrations (227). The antioxidant properties of date extracts help stabilize liver function and mitigate oxidative damage (228). Regular intake of Sukkari dates may contribute to long-term health improvements by reducing oxidative stress and supporting liver health (229). The glycemic indices (GI) of various date varieties grown in Saudi Arabia reveal significant variability, with a mean GI of 55.2 (SEM 7.7) across seventeen types. Notably, Sellaj and Maktoomi exhibited the highest GI, while Shaqra, Sukkary, and Sag'ai had the lowest values, indicating that certain varieties may be more suitable for individuals managing blood sugar levels (99). The GI of dates ranges from 42.8 to 74.6, highlighting the diversity among varieties (99). Sellaj and Maktoomi's high GI suggests they may cause a more significant increase in blood glucose levels compared to lower GI varieties like Shaqra, Sukkary, and Sag'ai (99). The ripeness of dates significantly affects their GI; for instance, Khalal stage dates have a higher GI compared to Tamer stage dates (100). This indicates that the consumption of dates at different maturation stages can influence glycemic responses, which is crucial for dietary planning (100). Despite the varying GIs, studies suggest that moderate consumption of dates may not adversely affect glycemic control in individuals with type 2 diabetes (98). The nutritional benefits of dates, including fiber and antioxidants, may contribute positively to overall health, potentially mitigating the impact of their sugar content (230).

**Figure 3:**
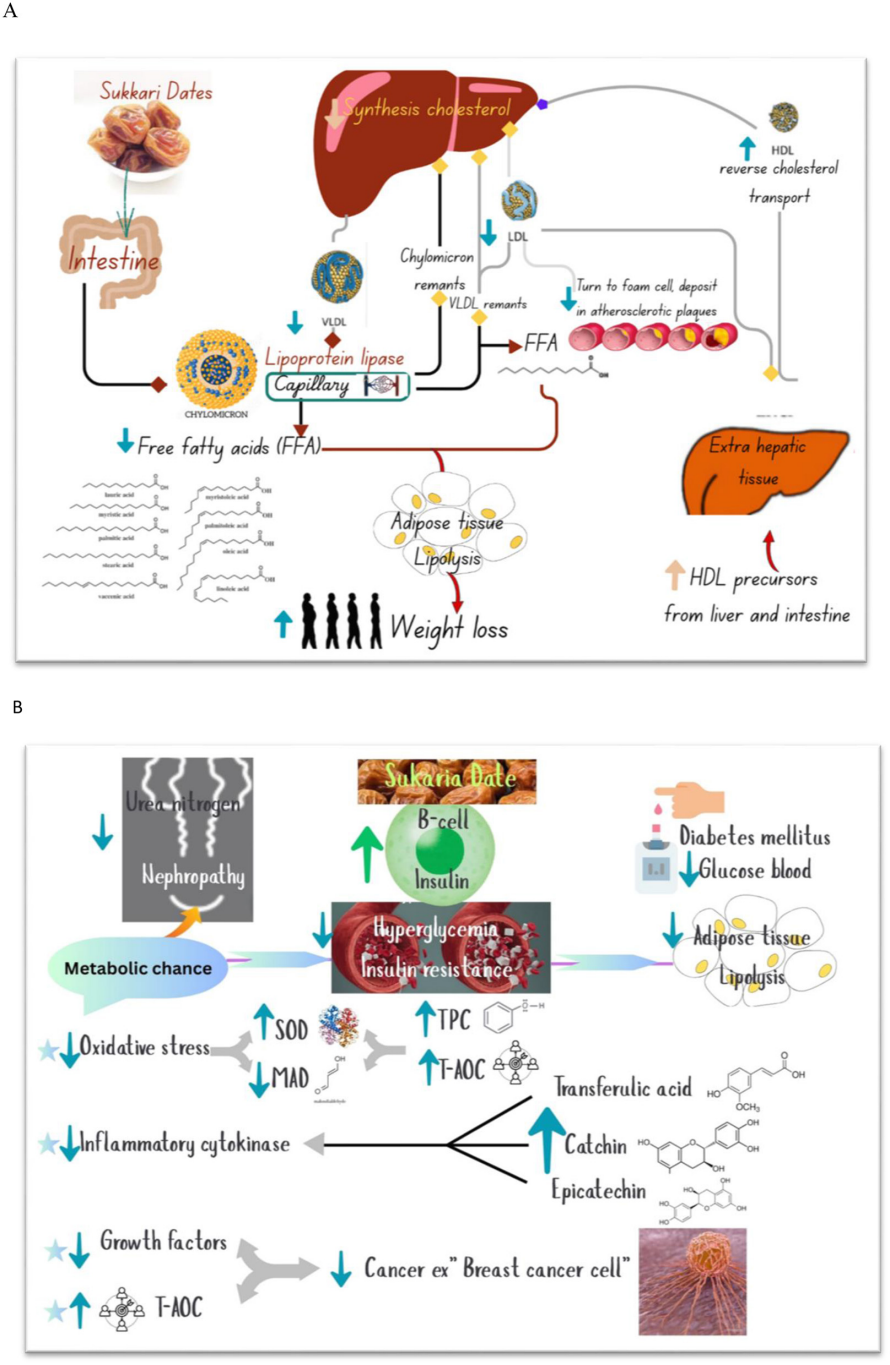
**(A, B)** Therapeutic effects of Sukkari dates. HDL, High-density lipoprotein; LDL, low-density lipoprotein; VLDL, very-low-density lipoprotein; FFA, free fatty acids. TPC, Total phenolic content; T-AOC, total antioxidant capacity; MDA, malondialdehyde; SOD, superoxide dismutase.

Sukkari dates have been shown to positively influence lipid profiles, contributing to decreased chylomicron levels, very low LDL, and overall cholesterol synthesis, while promoting weight loss and increasing HDL levels (Figure 3A). The consumption of dates can be part of a weight management strategy, as they are nutrient-dense and may replace higher-calorie snacks (231). While specific studies on chylomicrons are limited, the overall lipid-lowering effects suggest a potential for reduced chylomicron levels as part of improved metabolic health (232). Conversely, while Sukkari dates show promise in improving lipid profiles, the impact of other dietary components, such as phytosterols and low-carbohydrate diets, also plays a significant role in managing cholesterol levels and cardiovascular risk (233, 234).

The consumption of Sukkari dates has been shown to significantly enhance various biochemical parameters related to diabetes management and oxidative stress reduction. Research indicates that Sukkari dates can increase catechin, epicatechin, and total phenolic content, which contribute to improved antioxidant capacity and insulin secretion. Additionally, these dates have been linked to decreased levels of malondialdehyde (MDA), inflammatory cytokines, and growth factors, while also exhibiting potential anti-cancer properties, particularly against breast cancer cells (Figure 3B). Sukkari dates elevate catechin, epicatechin, and total phenolic content, enhancing total antioxidant capacity (T-AOC) and superoxide dismutase (SOD) levels (209, 235). The consumption of Sukkari dates promotes beta-cell function, leading to increased insulin levels, which is crucial for glucose regulation (236). Sukkari dates significantly lower MDA, a marker of oxidative stress, indicating reduced cellular damage (209, 235). The intake of these dates results in decreased inflammatory cytokines, which are often elevated in diabetic conditions (237). Sukkari dates have shown potential in reducing the proliferation of cancer cells, particularly breast cancer (209). Regular consumption leads to lower blood glucose levels, contributing to better diabetes management (210, 235). Conversely, while Sukkari dates exhibit numerous health benefits, excessive consumption may lead to increased caloric intake and potential weight gain, which could counteract their positive effects on diabetes management. Thus, moderation is key in their dietary inclusion.

## Food applications of Sukkari dates

**Figure 4:**
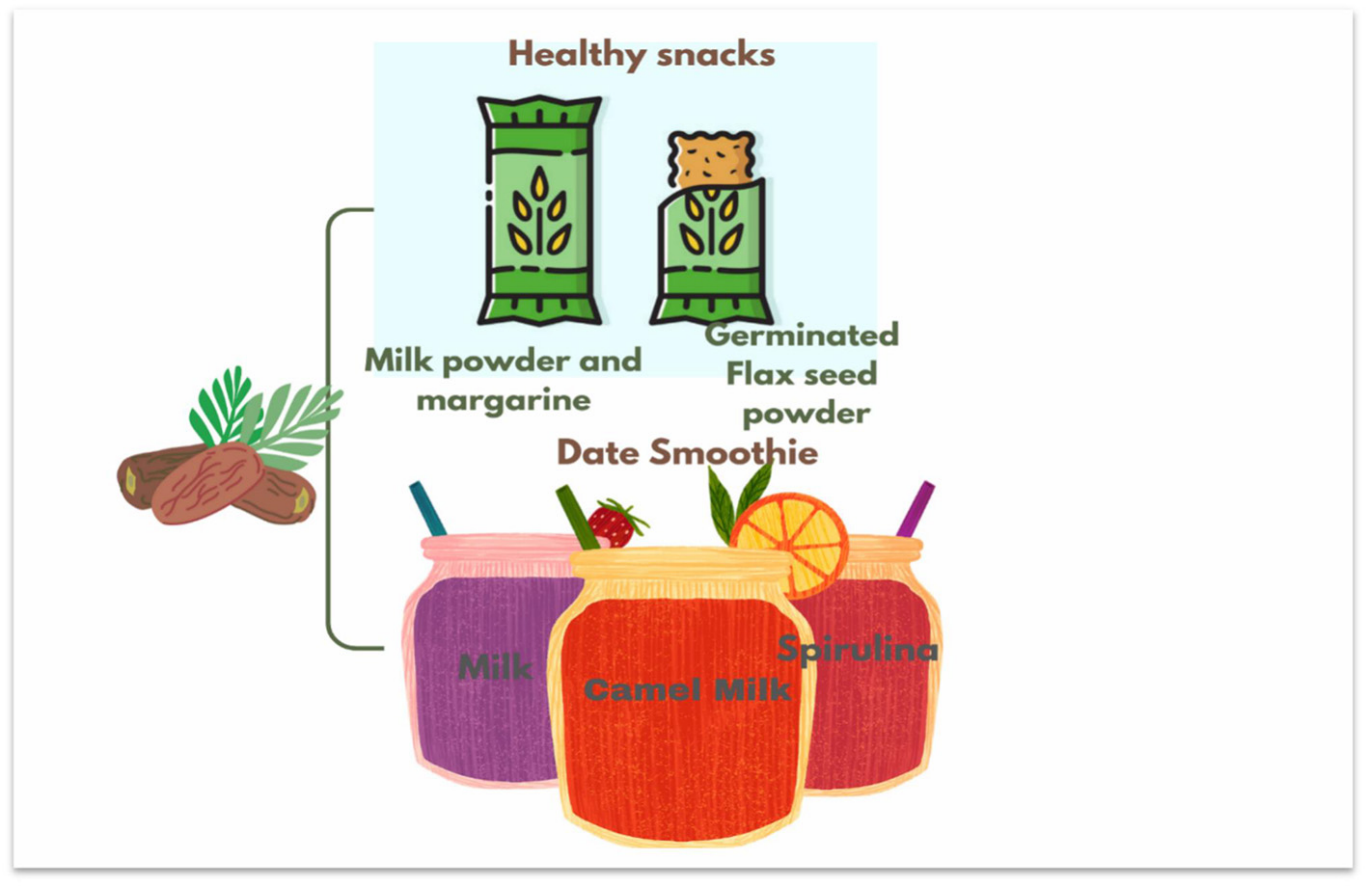
Key applications of Sukkari dates based on recent research.

Sukkari dates have diverse applications in enhancing nutritional profiles and developing innovative food products. Their rich nutrient composition makes them suitable for various formulations, particularly in health-focused markets (Table 8; Figure 4). Incorporating Sukkari dates into camel milk significantly enhances its nutritional profile, offering a richer source of vitamins and minerals, which is beneficial for consumers seeking nutritious dairy alternatives. This combination not only improves the milk's functional properties but also augments its health benefits, making it a valuable addition to the diet. Camel milk is naturally rich in vitamins C and E, and the addition of dates further enhances its antioxidant properties (238). This blend has shown potential in managing obesity and metabolic disorders, as evidenced by studies on rats where fermented camel milk with Sukkari dates improved lipid profiles and reduced body weight (11, 18). The combination of camel milk and Sukkari dates improves the antioxidant activity and total phenolic content, which are crucial for combating oxidative stress (11, 18, 239). This blend has shown potential in managing obesity and metabolic disorders, as evidenced by studies on rats where fermented camel milk with Sukkari dates improved lipid profiles and reduced body weight Aljutaily et al. (11, 18). Camel milk's therapeutic properties, such as treating gastrointestinal issues and boosting immunity, are complemented by the health benefits of dates, which include anti-inflammatory and cholesterol-lowering effects (238). The viability of probiotic strains in fermented camel milk is increased with the addition of dates, supporting gut health and providing a probiotic effect (11, 18). The incorporation of Sukkari dates enhances the sensory attributes of camel milk, improving flavor, consistency, and overall acceptability (11, 18, 239).

**Table 8:** Key applications of Sukkari dates based on recent research.

**Content reported in different studies**			
Algonaiman AND Alharbi (242) **Date Smoothie**	Enhance the nutritional profile and functional properties of camel milk	Camel milk was mixed with 10% Sukkari date paste and varying concentrations of oat beverage (0%, 25%, 50%, and 75%) to create different treatment groups. This mixture was then fermented at a temperature of 42 °C for approximately 3 h using the ABT-5 starter culture. The study measured microbial activity across the different treatments. Various tests were conducted to evaluate the nutritional content of the fermented products. This included measuring total phenolic content, antioxidant activity, and β-glucan levels. A sensory evaluation was conducted to assess the taste, color, aroma, and texture of the different treatments.	The results indicated that the treatment with 25% oat beverage (T2) exhibited higher microbial activity (2–7% increase). The study found that 25% oat beverage (T2) had a significant increase in total phenolic content and antioxidant activity, along with a β-glucan content of 0.05 g per 100 g dry weight. Sensory evaluation revealed that T2 was the most preferred treatment in terms of taste, color, aroma, and texture.
Parn et al. (240) **Healthy Snack**	Development of novel fruit bars by utilizing Nabtat Ali and Sukkari dates	The study utilized two popular date cultivars, Nabtat Ali and Sukkari. Fresh dates were peeled, halved, and pitted to obtain the pulp. The pulp (500 g) was pounded into a paste using a sterile mortar and pestle. This paste was boiled for about 10 min without adding any sugar, as dates are naturally sweet. Ingredients such as milk powder (100 g), margarine (50 g), citric acid (1 tablespoon), and a pinch of salt were added to the mixture. The mixture was boiled with water until a specific level of total soluble solids (TSS) was achieved, ensuring the product had minimal moisture and extended shelf life	Both types of date fruit bars were found to be rich in essential nutrients, including protein and fat, which contribute to their energy value. This makes them suitable for various age groups, including children and the elderly, who may benefit from easy-to-chew foods.
Alhomaid et al. (12) **Healthy Snack**	The study investigates germinated flaxseed powder's impact on date bars' nutrition	The research involved creating date bars by partially replacing Sukkari date paste with varying levels of GFP (0%, 4%, 8%, 12%, and 16%). The study evaluated the chemical composition of the date bars, including the analysis of minerals, fatty acid profiles, and amino acids.	The study found that blending date paste with GFSs led to a notable increase in protein content, particularly in bars containing 12% and 16% GFS, which had protein levels approximately 2.34 and 2.51 times higher than the unfortified control samples, respectively.
Jufri et al. (246) Date Smoothie	Developed a vitamin D-enriched date milk product aimed at preschool children aged 48–59 months. This product serves as a nutritional supplement to support their growth and development.	The research utilized a completely randomized design to evaluate the effects of different percentages of Sukkari date flesh added to liquid milk. The study specifically tested three formulations: F1 (10% date flesh), F2 (15% date flesh), and F3 (20% date flesh). The study analyzed various nutritional components such as water, ash, fat, protein, carbohydrates, zinc, iron, calcium, and vitamin D.	The addition of date flesh significantly influenced the water, protein, and carbohydrate content of the date milk. Specifically, the formulations with 10%, 15%, and 20% date flesh showed substantial differences in these components, indicating that the percentage of date flesh affects the nutritional profile of the drink. The selected formula, F2, which contained 15% date flesh, was found to be the most acceptable among the panelists based on hedonic rating and ranking tests. This formula received the highest scores for overall preference, indicating that it was favored for its taste and sensory attributes
Aljobair et al. (247) Date Smoothie	Development of nutritious drinks using date palm puree (Khalas and Sukkari) and spirulina	Different substitution rates of spirulina were tested in combination with the date puree. This method aimed to determine the optimal balance between the two ingredients to enhance nutritional value while maintaining sensory attributes. The nutritional value of the developed drinks was assessed, Sensory attributes of the drinks were evaluated through taste tests. The physicochemical properties of the drinks were analyzed.	The combination of date palm puree and spirulina significantly enhances the nutritional profile of the drinks. The sensory evaluation indicated that the drinks developed with varying substitution rates of spirulina were generally well-accepted by consumers. This synergy results in a drink that is not only tasty but also beneficial for health
Alfheeaid et al. (16) **Healthy Snack**	The study highlights that high-energy and protein bars made from Sukkari dates and fruit mixtures are beneficial for health-conscious individuals.	The study involved the formulation of two types of bars: a date-based bar (DBB) and a fruit-based bar (FBB). The DBB was made using 50% Sukkari date paste, while the FBB was created using a mixture of dried fruits, specifically 25% raisins, 12.5% figs, and 12.5% apricots, combined with other ingredients. The researchers conducted proximate analysis to determine the moisture, fat, ash, crude fiber, and protein content of both bars. The sugar content of both bars was analyzed. The study included an examination of the amino acid profiles. The mineral content of the bars was measured and evaluated the total phenolic content, total flavonoids, and total flavonols in both bars. They also assessed the antioxidant activity. Visual color parameters were measured. A sensory evaluation was conducted where panelists tasted both bars and provided feedback on their preferences.	The research found that the date-based bar (DBB) and fruit-based bar (FBB) have different nutritional profiles. However, the protein content was similar in both bars. The DBB had more sucrose. Additionally, the DBB was richer in several minerals such as calcium, copper, iron, zinc, manganese, and selenium but lower in magnesium, potassium, and sodium than in the FBB. Both bars contained essential and non-essential amino acids, but the DBB had higher levels of several essential amino acids. The fatty acid composition was similar in both bars, with palmitic acid being the most prevalent saturated fatty acid. The FBB exhibited higher total phenolic content, total flavonoids, and antioxidant activity compared to the DBB. Sensory tests indicated that panelists preferred the DBB over the FBB.
Hadi et al. (10) **Healthy Snack**	The study investigates the chemical composition of dates and their application in creating energy-rich snacks for athletes.	The research involved collecting date fruits from five different cultivars: Dekel Nour, Barhi, Zahdi, Khlas, and Sukkari. Once dried, the dates were ground using a grinder. This process transformed the whole fruits into a powdered form, making it easier to incorporate them into snack formulations. The study included a comprehensive analysis of the nutritional content of the different date cultivars. This involved measuring the percentages of total sugars, moisture, minerals, proteins, and fats.	The study noted that carbohydrates make up about 70% of the date's composition, primarily in the form of fructose and glucose, also contain significant amounts of minerals. Phosphorus was identified as the most abundant mineral, followed by magnesium, calcium, and proteins, making them suitable for athletes seeking effective energy sources.

Research has demonstrated the potential of Sukkari dates in creating fruit bars. These bars, devoid of added sugars, leverage the natural sweetness of dates, resulting in products that are high in protein, fat, and carbohydrates, making them appealing to health-conscious consumers (240, 241). Sukkari dates serve as a primary ingredient in high-energy and protein bars. These bars, containing a mix of dates and other fruits, provide significant caloric and nutritional benefits, making them ideal for athletes and active individuals (16). Innovative products such as vitamin D-enriched date milk and nutritious drinks combining date palm puree with spirulina have been developed. These products cater to specific demographics, such as preschool children, enhancing their dietary intake (263). The following sections outline key applications of Sukkari dates based on recent research.

The study by Algonaiman and Alharbi (242) explores the enhancement of camel milk's nutritional profile and functional properties through the incorporation of Sukkari date paste and varying concentrations of oat beverage. The results indicate that mixing camel milk with 10% Sukkari date paste and 25% oat beverage yields the most favorable outcomes in terms of microbial activity, antioxidant properties, and sensory attributes. The addition of Sukkari date paste and oats significantly boosts the total phenolic content and antioxidant activity, with the 25% oat beverage treatment showing a notable increase in iron content and a decrease in phytic acid (242). The incorporation of oats enhances the β-glucan content, which is beneficial for health, although further enrichment is suggested (242). The treatment with 25% oat beverage was preferred for its taste, color, aroma, and texture, indicating a successful combination of flavors and health benefits (242).

Camel milk is recognized for its potential in managing diabetes due to its insulin-like proteins, which may reduce blood sugar levels and improve glycemic control (243). While the study highlights the benefits of camel milk and its enhancements through Sukkari dates and oats, it is essential to consider that individual dietary needs and preferences may vary, and further research is necessary to fully understand the long-term health impacts of these combinations. The development of novel fruit bars utilizing Nabtat Ali and Sukkari dates has shown promising results in terms of nutritional value and consumer appeal. These date-based bars are rich in essential nutrients, including protein and fat, making them suitable for various age groups, particularly children and the elderly, who may require easy-to-chew foods (240). The bars leverage the natural sugars present in dates, eliminating the need for added sweeteners (240). Dates are known for their antioxidant and anti-inflammatory properties, enhancing the health benefits of the bars (241). Conversely, while these date-based bars are nutritious, some may argue that the high sugar content, even from natural sources, could be a concern for certain health-conscious consumers, particularly those managing blood sugar levels.

The study by Alhomaid et al. (12) highlights the nutritional enhancement of date bars through the incorporation of germinated flaxseed powder (GFS). The findings indicate that bars containing 12% and 16% GFS exhibited protein levels approximately 2.34 and 2.51 times higher than unfortified controls, showcasing the potential of GFS as a protein booster in snack formulations. The addition of GFS significantly elevates protein levels in date bars, making them more suitable for health-conscious consumers (16). Enhanced protein bars can provide essential amino acids, crucial for muscle repair and growth (244). In this regard, the incorporation of fried green lentil (Lens culinaris Medik.) does not adversely affect the texture or taste, which is vital for market success (245). While the focus on protein enhancement is significant, it is essential to consider the balance of other nutrients, such as carbohydrates and fats, to ensure a well-rounded snack option. This holistic approach can cater to diverse dietary needs and preferences.

The development of a vitamin D-enriched date milk product for preschool children, as explored by Jufri et al. (246), highlights the nutritional benefits of combining milk with date flesh. This innovative product not only enhances the dietary intake of essential nutrients but also addresses the sensory preferences of young children. The study identified that varying percentages of date flesh significantly influenced the nutritional profile, particularly in water, protein, and carbohydrate content, with the 15% formulation (F2) being the most favored by taste tests. Dates are rich in essential minerals such as potassium, magnesium, and iron, contributing to overall health and development (35). The combination of date palm puree and spirulina in the development of nutritious drinks significantly enhances their nutritional profile, offering a tasty and health-beneficial option for consumers. The synergy between these ingredients results in a drink that is not only rich in essential nutrients but also well-received in terms of sensory attributes. This combination leverages the high nutritional value of both date palm puree and spirulina, making it a promising functional beverage. Date palm puree, particularly from Khalas and Sukkari cultivars, provides a rich source of natural sugars, vitamins, and minerals, contributing to the drink's overall nutritional value (247). Spirulina is renowned for its high protein content, essential amino acids, vitamins, and minerals, which significantly boost the nutritional profile of the drink. It is particularly noted for its health benefits, such as enhancing immunity and providing essential nutrients like γ-linolenic acid (76, 248, 249). The combination with date palm puree enhances these benefits, providing a drink that supports overall health and wellbeing (247). The study by Alfheeaid et al. (16) emphasizes the nutritional benefits of high-energy and protein bars made from Sukkari dates and fruit mixtures, revealing distinct nutritional profiles between date-based bars (DBB) and fruit-based bars (FBB). While both bars share similar protein content, the DBB is richer in minerals and essential amino acids, whereas the FBB boasts higher antioxidant properties. The study by Hadi et al. (10) highlights the nutritional benefits of dates, particularly their suitability for athletes seeking energy-rich snacks. Dates are composed of approximately 70% carbohydrates, primarily fructose and glucose, making them an excellent source of quick energy. Additionally, they contain significant minerals, with phosphorus being the most abundant, followed by magnesium and calcium, which are essential for athletic performance and recovery. Carbohydrates comprise about 70% of dates, mainly as simple sugars (fructose and glucose) (10). Dates are rich in phosphorus, magnesium, and calcium, contributing to energy metabolism and muscle function (10, 53). Dates provide B-complex vitamins and vitamin C, supporting overall health and energy levels (53). They contain bioactive compounds that may enhance recovery and reduce oxidative stress (241). Conversely, while dates are nutrient-dense, they are low in protein, which may necessitate the addition of other protein sources in energy bars to meet athletes” dietary needs (16).
